# Exploring The Sensory and Aroma Characteristics of Rakı Through Check-All-That-Apply and Consumer Preference Approaches

**DOI:** 10.3390/foods15081321

**Published:** 2026-04-10

**Authors:** Merve Darıcı

**Affiliations:** Department of Food Engineering, Faculty of Engineering, Çukurova University, 01250 Sarıçam, Adana, Türkiye; mdarici@cu.edu.tr

**Keywords:** rakı, check-all-that-apply, descriptive analysis, sensory profile, aroma profile, consumer preference, preference mapping, distilled beverages, stir bar sorptive extraction

## Abstract

Rakı, a traditional distilled beverage produced from grapes, holds significant economic importance in Türkiye; however, comprehensive consumer-focused sensory research remains limited. This study aims to determine the aroma profile, sensory characteristics, and consumer preferences of commercial rakı to guide producers in aligning product characteristics with consumer expectations. Nine commercial rakı samples were evaluated. The aroma composition was analyzed using SBSE-GC-MS. Sensory attributes were assessed by a trained panel through descriptive analysis (DA) and by 100 consumers utilizing the Check-All-That-Apply (CATA) method alongside a liking test. Eighty-one aroma compounds were identified, predominantly the phenylpropanoids trans-anethole and estragole, with monoterpenes and sesquiterpenes dominating the secondary profile. Integrating instrumental data with DA evaluations suggests that anethole and sesquiterpenes likely contribute to the attributes related to visual coating, body, creamy, mastic, persistency, and complexity. Consumer profiling revealed two distinct preference groups. Older, frequent consumers preferred complex, high-alcohol profiles with trigeminal harshness and visual glass coating, whereas younger, casual consumers preferred smoother rakı with a traditional white appearance, reacting negatively to “boiled aniseed” flavors and the yellowish tint of oak-aged versions. The CATA technique effectively distinguished these profiles. To enhance overall product quality, producers should eliminate “boiled” defects and adjust sensory profiles: complex products for experienced consumers and visually traditional, smooth profiles for younger consumers. According to current knowledge, this is the first study to employ the CATA method alongside consumer profiling and preference mapping in the sensory evaluation of rakı.

## 1. Introduction

Rakı, a traditional grape-based distilled beverage with a long cultural background, is the most widely produced and consumed distilled beverage in Türkiye. With an annual production volume of 3.5 million tons [1], Türkiye is the world’s seventh largest grape producer and a leading vine grower in terms of both area and yield. The annual production of rakı in Türkiye stands at around 39.8 million liters [2], highlighting its status as a high-value-added product of great cultural significance. Furthermore, Türkiye exports approximately 5.6 million liters of rakı annually [2].

Rakı is produced by distilling Suma (grape distillate) with aniseed (specifically *Pimpinella anisum* L.) in a traditional copper pot still with a maximum capacity of 5000 L, in accordance with the Turkish Food Codex Communique on Distilled Alcoholic Beverages [3]. Notably, it was granted Protected Designation of Origin (PDO) status by the Turkish Patent and Trademark Office in 2009 [4]. Turkish regulations establish specific criteria to standardize and maintain the quality of rakı. Accordingly, rakı must be produced entirely in Türkiye, and at least 65% of its distillates (Suma) must derive from fresh grape or raisin-based distillates. Furthermore, Suma, a grape-based distillate, is required to be distilled at up to 94.5% alcohol by volume using column stills to retain the characteristic volatiles of the grapes. During the subsequent copper pot still distillation processes, *Pimpinella anisum* L. is added as the primary essential oil source, giving the beverage its characteristic flavor. The anethole content derived from aniseed must be at least 800 mg per liter in the final product. Additionally, the minimum alcoholic strength by volume of rakı is mandated at 40%, and the total volatile compounds content must be equal to or exceed 100 g per hectoliter of 100% volume alcohol [3,4,5,6].

Aroma is a key component in creating the characteristic sensory profiles of distilled alcoholic beverages. Rakı is defined as a product able to stimulate multiple senses through its complex aromatic composition [7,8]. Previous studies have also indicated that rakı possesses a rich aromatic profile, in which anethole and estragole are the major components derived from aniseed [5,6,7,8]. The aroma composition and sensory characteristics of rakı are influenced by numerous variables, including grape variety, the selection between fresh grapes or raisin-based distillate, the quality and quantity of aniseed (fresh or stored), and the processing steps, including fermentation, distillation technique, the number of distillations, and maturation [6].

To characterize the complex rakı matrix, robust aroma profiling is required. Analyzing the aroma compounds in distilled spirits presents a significant analytical challenge due to the high ethanol content, which can affect traditional extraction methods such as liquid–liquid extraction and cause fiber competition in techniques like solid-phase micro-extraction (SPME) [9]. Furthermore, the high concentration of aniseed essential oil makes traditional solvent-based extractions for such an oily and high-alcohol matrix highly challenging. To overcome these limitations, solvent-free micro-extraction techniques are preferred [10]. Among these, stir bar sorptive extraction (SBSE) stands out as an ideal technique for high-alcohol distilled beverages. Comparative studies have demonstrated that SBSE yields significantly higher recoveries of volatile compounds than SPME due to the substantially larger amount of sorbent applied [11,12]. Moreover, SBSE requires no additional solvents and successfully manages the complex interactions between ethanol and essential oils [12]. When coupled with gas chromatography–mass spectrometry (GC-MS), the SBSE-GC-MS approach ensures high sensitivity and robust quantification of the aroma compounds [10]. Despite the importance of advanced instrumental aroma profiling, comprehensive studies combining it with consumer perception data remain highly limited for rakı [5,7,8].

Descriptive analysis (DA) is a method generally used to describe the sensory properties of a product in detail or to compare the sensory differences among multiple products [13]. DA is often used for assessing the closeness of a new product to the intended target product, evaluating the suitability of developed products, and assessing consumer perceptions. Quantitative descriptive analysis (QDA), one of the most widely used DA techniques, is utilized to evaluate sensory attributes [14]. Despite the validity of DA methods when performed by trained panelists, they require considerable time and effort [15,16]. Consequently, consumer-oriented descriptive sensory analyses such as Flash Profile, Napping, and Check-All-That-Apply (CATA) have recently gained interest as new and rapid methods [17]. 

The Check-All-That-Apply (CATA) sensory approach is a rapid and effective descriptive analysis technique used to define product characteristics based on consumer perceptions, offering significant cost and time efficiency compared to traditional descriptive analysis [18]. It is widely recognized for its simplicity, allowing untrained panelists to identify and differentiate food and beverage products [19,20]. In this technique, assessors use CATA questions consisting of a predetermined list of descriptors from which they select any terms they consider suitable to characterize a product [21]. CATA enables all panelists, regardless of training, to efficiently select descriptors considered suitable for characterizing each product, thus avoiding the necessity for repeated analysis. However, similar to the other consumer-oriented methods, the CATA method requires at least 80 participants to ensure the reliability of the analysis and to reduce measurement inconsistencies arising from a lack of sensory training [22]. Non-parametric tests, such as the chi-square distribution and correspondence analysis (CA), are applied to evaluate the results statistically and generate visual maps [23]. Furthermore, CATA is highly suitable for simultaneous application [24,25] with liking/acceptability tests (such as the hedonic scale), which represent important and simple consumer assessments [26,27]. By evaluating CATA together with liking scores, preference maps are created, enabling researchers to effectively link sensory descriptors to consumer preferences and establish consumer targets [27].

Recognizing that the future of rakı and its economic value are significantly influenced by effective marketing, it is crucial to understand consumer perception by linking aroma profiling with sensory characteristics. Recent research on sensory descriptive evaluation has focused on the Check-All-That-Apply (CATA) technique, with studies assessing a wide range of product groups [28,29,30,31,32,33,34,35]. However, there are limited studies regarding the use of CATA and detailed consumer research on the sensory characteristics of rakı, with most existing research focusing only on the sensory profile [6] or aroma profiles [5,7,8]. Addressing this gap, the present study introduces a novel approach: as far as is known, it applies the advanced SBSE-GC-MS technique to rakı for the first time, while pioneering the first consumer-based CATA study combined with consumer profiling and preference mapping for this beverage. Therefore, this study has several key objectives: (1) to determine the aroma profile of commercial rakı products, (2) to identify the sensory descriptors based on DA and the CATA technique, and (3) to map consumer profiles and preferences, aiming to guide producers’ product development decisions by revealing which descriptors primarily influence consumer preferences.

## 2. Materials and Methods

### 2.1. Rakı Samples

Commercial rakı samples (*n* = 9, Table 1) were selected based on the quality categories and high purchase volumes from retail outlets. The quality categories were determined by factors including Suma source, distillate source and proportion, middle-cut proportion, the addition of heads and tails, the number of pot still distillations, and aniseed quantity. Particular attention was given to ensuring the representation of high-quality categories and a wide range of production applications available in the market. All samples were stored at 20 °C in a dark storage unit. Furthermore, all samples were sealed with standard non-refillable closures and had been kept in stainless steel tanks for at least one month prior to bottling.

### 2.2. General Analysis

Analyses of alcoholic strength by volume and sugar levels were carried out according to the OIV reference methods for spirituous beverages of vitivinicultural origin [36]. The determination of alcoholic strength by volume was performed using Anton Paar Alcolyzer (DMA 4500M-Alcolyzer ME, Anton-Paar, Graz, Austria) with Near-Infrared (NIR) spectroscopy, in accordance with the OIV reference method (OIV-MA-BS-08: R2009). Sugar levels were determined via HPLC analysis using a Shimadzu LC-20AT instrument (Shimadzu Corporation, Kyoto, Japan) equipped with a Refractive Index (RID-10A) detector, according to the OIV reference method (OIV-MA-BS-11: R2009) [36]. Analyses were performed in triplicate, as shown in Table 1.

### 2.3. Analysis of Trans-Anethole and Estragole Compounds/Direct Injection to GC-FID/MS

Trans-anethole and estragole were determined via direct injection using GC-FID/MS according to the European Commission Reference Method [37]. DL-Menthol was used as an internal standard. A 2 mL aliquot of the sample was mixed with 2 mL of an internal standard solution (0.19 g/L in 45% ethanol) in a 20 mL flask. Subsequently, 1 µL of the above mixture was injected into the GC. The GC-FID/MS conditions, column specifications, and identification and validation parameters of this method were previously reported in detail by Darıcı, Bergama, and Cabaroglu [7]. The GC equipment used was an Agilent 6890N (Agilent Technologies Co., Santa Clara, CA, USA) coupled with an FID. A CP-WAX 57 CB capillary fused silica column (polyethylene glycol stationary phase, 60 m × 0.25 mm i.d. with a 0.4 µm film thickness; Agilent, Netherlands) was employed. Injections were made in split mode (split ratio of 40:1). The injection temperature was set to 230 °C, and the oven was programmed isothermally at 180 °C for 15 min. The FID temperature was 230 °C (H_2_: 35 mL/min and air: 400 mL/min). The carrier gas was helium with a flow rate of 2 mL/min. Concentrations were calculated with respect to the internal standard from the relative response factors (RF) by using the FID signal. The results were expressed in mg/L.

### 2.4. SBSE-GC-MS Analysis

The stir bar sorptive extraction (SBSE) technique, as described by Buck et al. [12], was applied with modifications for the analysis of minor aroma compounds. The rakı samples were diluted individually 1:1 (*v*/*v*) in water into 20 mL glass headspace vials. Subsequently, 4-nonanol (15 µg) and DL-menthol (69 µg) were added to the samples as internal standards. The diluted mixture was stirred at room temperature for 30 min. Thereafter, a stir bar (10 mm length, 0.5 mm coating thickness; Gerstel GmbH, Mülheim an der Ruhr, Germany) coated with polydimethylsiloxane (PDMS) was added to the diluted sample. Teflon-coated crimp caps were used to seal the vials, and the mixture was stirred at room temperature for 2 h at 800 rpm.

After being stirred, the stir bar was removed, gently dried with a lint-free tissue and then transferred into a glass thermal desorption tube for GC–MS analysis. An Agilent 6890N gas chromatograph coupled with an Agilent 5975B INERT MSD mass spectrometer (Agilent Technologies Co., Santa Clara, CA, USA), equipped with a Gerstel Multi-Purpose sampler (Gerstel GmbH, Mülheim an der Ruhr, Germany), was used. The stir bar was placed in a glass thermal desorption tube within a Thermal Desorption Unit (TDU). The TDU was initially set to 30 °C (initial time: 0.5 min), then increased to 280 °C at a rate of 100 °C/min and held at this temperature for 10 min. The extracted volatiles were transferred to the injection system/programmed temperature vaporizer (CIS3) and cooled with 50% ethyl alcohol solution using a Universal Peltier Cooling (UPC) system (Gerstel GmbH, Mülheim an der Ruhr, Germany). The CIS injector was initially kept at 30 °C, and after an equilibration time of 0.05 min, the temperature rapidly increased to 280 °C at a rate of 10 °C/min; we held this temperature for 5 min. The split ratio was 20:1, and the CIS total flow of helium was 32.0 mL/min.

An HP-5MS capillary column (30 m × 0.25 mm × 0.25 μm, Agilent, Waldbronn, Germany) was employed to separate the aroma compounds. The initial temperature of the column was set to 40 °C, with a 4-min hold. After the hold, the temperature was increased by 2 °C per minute to 90 °C, then by 3 °C per minute to 130 °C, and finally by 4 °C per minute to 240 °C, where it remained constant for 5 min. Helium was used as a carrier gas at a constant flow rate of 1.4 mL/min. Mass spectra were generated in full scan mode (*m*/*z* range 35–250, EI 70 eV).

The aroma compounds were identified by comparing their retention indices and mass spectra on the HP5 column with data from a commercial spectral database (NIST MS 2.0, W10N14, W12N20) and the instrument’s internal library, which was compiled from previous laboratory studies. The injection of chemical standards into the GC-MS confirmed several of the identified compounds. The retention indices for the compounds were calculated using an n-alkane series. Specifically, the identification criteria were set to a minimum mass spectral matching factor ≥ 80%, and a maximum permitted difference of ±15 units between the calculated Linear Retention Index (LRI) and the literature/database LRI. After identifying the aroma compounds, the internal standard method was employed for tentative quantification. Subsequently, mean values were calculated based on the GC analyses conducted in triplicate.

### 2.5. Sensory Evaluation

This study was carried out in accordance with the Declaration of Helsinki for studies on human subjects. The sensory study protocols were approved by the Ethics Committee of Çukurova University (No 1514096). All assessors were properly informed about the research objectives and provided written consent for their participation.

#### 2.5.1. Sample Preparation and Serving

The rakı samples (Table 1), which were gathered from the distillers in Türkiye, were presented in tulip-shaped wine tasting glasses [38], each labeled with a random three-digit code. The glasses used for the sensory evaluations featured scale marking lines at 25 mL and 50 mL volume. Samples (25 mL) were served randomly at 20 °C. While rakı is typically consumed with tap water added at a 1:1 or 1:2 ratio depending on the consumer’s preference, all samples in this study were strictly diluted at a standard 1:1 ratio during the evaluation to ensure consistency. Specifically, 25 mL rakı samples were mixed with 25 mL of drinking water at 20 °C to be served during the evaluation.

To minimize ethanol intake and ensure participant safety, assessors were instructed to take only small sips sufficient to evaluate the sensory attributes and to expectorate the remaining liquid into the provided spittoons. A two-minute break was taken between each sample, during which water and plain crackers were served as palate cleansers.

The serving order for the Check-All-That-Apply (CATA) panel, involving 100 untrained assessors, was generated using the Design of Experiments (DoE) module in XLSTAT (Addinsoft, New York, NY, USA, version 2021.4.1), based on a Randomized Complete Block Design. To minimize panelist fatigue while maintaining statistical balance, each of the nine samples was evaluated by all assessors across two separate sessions. In this setup, five samples were presented in the first session and four samples in the second, ensuring that every assessor evaluated the full set while minimizing first-order and carry-over effects.

#### 2.5.2. Panel

The trained panel consisted of four females and four males aged 25–52 years, all of whom were academic personnel from the Food Engineering Department of Çukurova University. These personnel had a minimum of 300 h of training according to ISO 8586:2023, ISO 5496:2006, and ISO 3972:2011 [39,40,41] along with extensive experience in descriptive analysis. They had regularly participated in sensory research at the university.

The untrained panel consisted of one hundred consumers (*N* = 100) recruited through the consumer database of the Food Engineering Department. Before conducting the CATA evaluation, demographic data were obtained via an information form asking about their age, gender, and frequency of rakı consumption, which was categorized as rarely, several times a year, several times a month, or once a week.

#### 2.5.3. Descriptive Analysis (DA)

A trained sensory panel evaluated the samples, using generic descriptive analysis (GDA) as described by Lawless and Heymann [13]. GDA was carried out across eight sessions. In the first session, reference standards were prepared for each attribute based on the lexicon list created in a previous study [6]. During these sessions, the assessors familiarized themselves with the attributes from the lexicon. In the second session, the panelists developed descriptive terms for the rakı samples during tasting. In the third and fourth sessions, different aroma and taste standards were presented again and discussed by the assessors.

As a result, three appearance (white color, yellow color, visual coating), sixteen aroma (pungent, fresh aniseed, boiled aniseed, Suma odor, grape, raisin, mastic, menthol, resin/fresh, spicy, black pepper, coriander, clove, bitter almond, dried flower, wood/oak), nine retronasal aroma (Suma, grape, raisin, fresh aniseed, boiled aniseed, mastic, menthol, spicy, dried flower), and nine taste/mouthfeel terms (sweetness, bitterness, astringency, body, alcohol burning, creamy, throat harshness, complexity, persistency) were selected for the descriptive analysis.

In the fifth and sixth session, the assessors performed a training evaluation with each attribute. The seventh and eight sessions, which served as formal (data collection) sessions, were carried out with two repetitions. The assessors evaluated the intensity of each attribute on a 15 cm unstructured line scale anchored by “low” and “high” intensities. In the formal sessions, four samples were served in the morning, while five samples were served in the afternoon sessions. Two sessions were held each day, one in the morning and one in the afternoon. Every sample was served twice.

#### 2.5.4. Check-All-That-Apply (CATA)

##### Determination of CATA Questions

To develop the descriptors for the CATA questions, the attributes identified in the GDA study were utilized. The trained panel gathered to simplify the descriptive terms from the GDA study to enhance their clarity and practicality for consumers. While the DA attributes consisted of specific, expert-level terminology, the CATA descriptors were deliberately adapted to reflect a naive consumer vocabulary. The panel reached a consensus on the descriptors to be used in the CATA question list. A total of 41 descriptive terms were selected for the CATA questions, comprising seven for appearance, sixteen for aroma, and eighteen for taste/flavor. The CATA question list is presented in Appendix A.2. The ballot consisted of three columns, each representing a group of sensory characteristics. Furthermore, to facilitate rapid comprehension, all descriptive terms were arranged in columns according to the temporal order of perception. Consumers tend to spend less time responding to CATA questions when the terms are presented in a fixed order. Additionally, research suggests that using a fixed logical order or an alphabetical order has minimal impact on participants’ selections [24,42].

##### CATA Procedure

Prior to sample presentation, the analysis protocol and the CATA questionnaire (Appendix A.2) were verbally explained to each participant (Appendix A.2). All consumers were requested to review the descriptors to ensure that they fully understood the CATA questions and all the terms used. Additionally, the descriptors used in the form and their corresponding reference list (Supplementary Table S1) were presented to the consumers to facilitate accurate identification of the sample attributes. Consumers were then asked to select all the relevant attributes from the CATA form to characterize the sensory properties of the served rakı samples. Participants were instructed to assess the samples based on appearance, aroma, and taste/flavor.

Furthermore, a liking test was administered to the consumers following the CATA analysis. The liking test employed a 9-point hedonic scale, ranging from “extremely liked” to “extremely disliked” [43]. Following the completion of the CATA tasks, participants provided their liking rating for each sample.

### 2.6. Statistical Analysis

Data analysis was conducted using the XLSTAT program (Addinsoft, New York, NY, USA, version 2021.4.1). Statistical significance was set at an alpha level of 0.05 (*p* < 0.05) for all analyses unless otherwise stated. To identify the differences among the samples, one-way analysis of variance (ANOVA) was utilized to analyze aroma composition, while two-way ANOVA was applied to the sensory data from the DA study. Additionally, panel performance (repeatability and discrimination) was monitored during the training sessions. Principal component analysis (PCA) was also performed to visualize these differences. In the assessment of demographic data, Agglomerative Hierarchical Clustering (AHC) was performed using Ward’s method to group consumers exhibiting similar liking trends. Subsequently, a chi-square test was applied to the demographic data. Two-way ANOVA was then conducted on the clustered liking data to assess the differences in preferences among the identified clusters.

For the CATA data, the counts of checked attributes across all consumers were initially tabulated to generate a contingency table. Correspondence analysis (CA) was then applied to the CATA data to evaluate how consumers described rakı samples. Cochran’s Q test was performed to determine statistically significant differences in CATA attributes across products. While the standard significance level of *p* < 0.05 was used to identify primary discriminators, descriptors with *p*-values below 0.20 in Cochran’s Q test were also retained in the CA to ensure that all potentially relevant sensory attributes were captured. Significant differences between the samples were evaluated using Sheskin’s multiple comparison test.

Penalty analysis was conducted by integrating CATA descriptors with overall liking scores to determine the descriptors that positively or negatively influence preferences. To determine the significance of these attributes, two-sample *t*-tests were performed to compare the differences in average preference scores when an attribute was checked (Present) versus when it was unchecked (Absent) by consumers. Finally, external preference mapping analysis (PrefMap) was employed to establish the relationship between the grouped liking data and the CATA data. Consumer cluster liking was then overlaid onto the PrefMap (XLSTAT version 2021.4.1) using the vector model, which was determined to be the most appropriate regression model for the obtained data.

## 3. Results and Discussion

### 3.1. Aroma Profile

The aroma composition of the commercial rakı products is presented in Supplementary Table S2. In the products, a total of 81 aroma compounds were identified, including 11 esters, 9 monoterpene hydrocarbons, 1 monoterpenoid alcohol, 6 oxygenated monoterpenes, 17 phenylpropanoid derivatives, 23 sesquiterpene hydrocarbons, 2 sesquiterpene alcohols, 2 aromadendrane sesquiterpenoids, 3 volatile phenols, 2 higher alcohols, 2 aldehydes, 1 alkene, and 1 furan. Across all products, the total concentration of aroma compounds varied between 1121 and 2235 mg/L. Trans-anethole and estragole were the most dominant compounds derived from aniseeds, with concentrations ranging between 1075 and 2126 mg/L and between 16 and 56 mg/L, respectively. Their odor threshold values are 0.073 and 0.016 mg/L, respectively, and they have anise-like odors [44]. The direct use of aniseed (*Pimpinella anisum* L.) in rakı processing is one of the key distinctions between rakı and other aniseed-flavored distilled beverages. According to my previous study on 37 commercial rakı samples [6], trans-anethol and estragole concentrations ranged between 1010.0 and 2060.0 mg/L and 19.5 and 58.6 mg/L, respectively. The trans-anethole concentration must be 800 mg/L as a minimum according to the Turkish Distilled Alcoholic Beverage Regulation [3] and Protected Designation of Origin (PDO) specifications. It is a crucial aroma component as it defines rakı’s unique flavor. The sensory quality of rakı is significantly influenced by the quantity of aniseed used in the production process.

**Figure 1:**
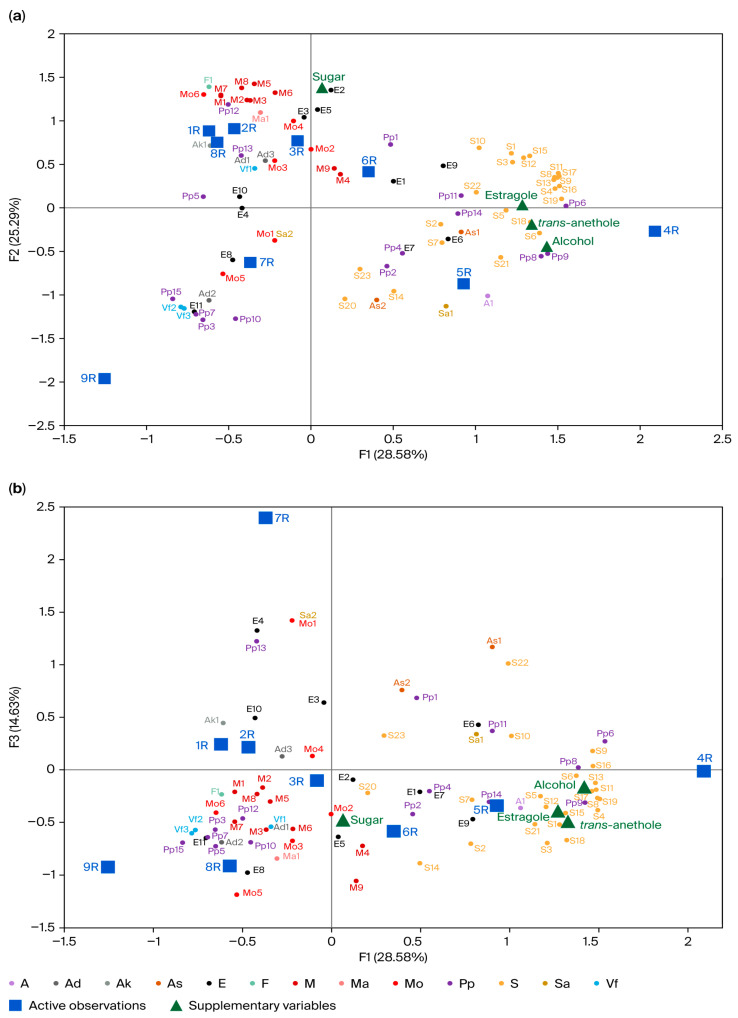
Principal component analysis of the aroma composition of rakı. (**a**), PC1 and PC2 dimensions; (**b**), PC1 and PC3 dimensions. Active variables: The aroma compounds are categorized as follows: A: higher alcohol; Ad: aldehyde; As: aromadendrane; E: ester; F: furan; M: monoterpene; Ma: monoterpene alcohol; Mo: oxygenated monoterpene; Pp: phenylpropanoid; S: sesquiterpene; Sa: sesquiterpene alcohol; and Vf: volatile phenol. The aroma compounds and their corresponding codes are given in Appendix A.1. Supplementary variables: trans-anethole, estragole, alcohol and sugar contents.

The distribution of aroma composition is visualized using principal component analysis (PCA) with three dimensions, as shown in Figure 1. 

In this PCA model, the aroma compounds obtained from the SBSE-GC-MS analysis were used as active variables, whereas the major aroma compounds (trans-anethole and estragole) analyzed via direct injection GC-FID/MS, along with the alcohol and sugar contents, were included as supplementary variables (Figure 1).

As shown in Figure 1, PC1 explains 28.6% of the variance, PC2 represents 25.3% of the variance, PC3 accounts for 14.6% of the variance, and PC4 explains 12.2% of the variance, totaling up to 80.7% of the variance. PC4 was excluded for the purpose of simplicity. Principal components PC1, PC2, and PC3 separated the products into three groups, as shown in Figure 1. Products R4, R5, and R6 are positioned on the positive side of the PC1 component, showing a strong correlation with phenylpropanoids (Pps) and sesquiterpenes (Ss). Specifically, 4R, which was produced from 100% fresh grape distillate (Suma) via a triple-distillation process and had the highest alcohol strength (47.5% ABV), exhibited the maximum concentration of phenylpropanoid derivatives and sesquiterpenes. Products 1R, 2R, and 3R were primarily characterized by monoterpenes (Ms). Furthermore, 8R and 9R are located in the negative quadrants of the PC1 and PC3 biplot (Figure 1b). These samples were characterized by volatile phenols (Vfs), due to their short-term treatment in oak barrels. Additionally, high levels of oxygenated monoterpenes (Mos) were observed in 8R and 9R, with 9R exhibiting the highest concentration (6245 μg/L) among all products. This is closely associated with the oak barrel maturation process specified in the production parameters of these samples. Barrel maturation likely promotes the oxidation of terpene hydrocarbons into oxygenated derivatives, such as D-carvone, which is the dominant compound in 9R.

Quantitative evaluation of the identified volatile compounds demonstrated substantial concentration ranges, which significantly influenced product characterization. Products with elevated anethole content, particularly 4R and 5R, exhibited higher quantities of sesquiterpenes (Ss) than other products. For instance, γ-himachalene (S11), a prominent sesquiterpene, reached its highest level in 4R (9830 µg/L) and maintained a high concentration in 5R (5672 µg/L), which corresponds to an elevated anise-like aroma intensity. In contrast, the other products, especially 1R and 2R, were primarily defined by monoterpenoid compounds. Notably, for 1R and 2R, monoterpene hydrocarbons, including D-limonene (6936 µg/L and 3504 µg/L, respectively), and monoterpenoid alcohols, such as linalool (1347 µg/L and 1561 µg/L, respectively), contributed as the primary aroma components.

Furthermore, aside from trans-anethole, estragole, and γ-himachalene, other aroma compounds were also present in dominant concentrations. For example, p-anisaldehyde (Pp3) showed significant variation, ranging from 576 µg/L in R2 to 14,399 µg/L in 9R. Linalool (Ma1), a significant monoterpenoid alcohol, varied from 182 µg/L in 7R to 1751 µg/L in 8R, giving floral characteristics. Ethyl-2-methyl butyrate (E2), among the esters, varied from 76 µg/L (9R) to 479 µg/L (3R), providing fruity aromas. These results align with our previous study on rakı aroma [7] which identified 51 volatile and 19 odor-active compounds, establishing that the primary contributors to the aroma of rakı are anethole, estragole, linalool, ethyl-2-methyl butyrate, γ-himachalene, and p-anisaldehyde.

The distribution of esters among the samples was primarily related to the Suma source, with 100% fresh grape Suma samples (4R, 8R, and 9R) exhibiting the highest concentrations (4799–6273 µg/L). Interestingly, 5R, produced from 100% raisin Suma, was uniquely characterized by the highest individual concentration of ethyl hexanoate (1081 µg/L). Ylmaztekin et al. [8] reported the identification of 43 aroma compounds, with trans-anethole, valencene, estragole, and cis-anethole comprising the primary compounds found in rakı. They also identified esters of fermentative origin, such as ethyl octanoate and ethyl decanoate.

### 3.2. Sensory Profile

A trained panel of eight assessors evaluated the sensory attributes of rakı by using generic descriptive analysis (GDA). The assessors evaluated rakı based on three appearance, sixteen odor, two taste, five mouthfeel, and eleven flavor attributes using a 15 cm line scale. Supplementary Table S3 shows the mean values of the intensities of each attribute for each sample, as assessed by the trained panel. Significant differences (*p* < 0.05) were detected in nine attributes describing the rakı samples: color, pale yellow, visual coating, coriander, wood/oak, astringency, body, creamy, and persistency attributes. These significant attributes primarily reflect the two critical production variables: aniseed content and oak maturation.

Specifically, attributes such as visual coating, body, creamy, and persistency are related to the quantity and quality of aniseed (primarily trans-anethole), which enhances viscosity and the characteristic whitening effect. Conversely, pale yellow, wood/oak, and astringency are exclusively driven by the extraction of phenolic compounds during short-term oak barrel aging, clearly differentiating these samples from traditional rakı products.

**Figure 2:**
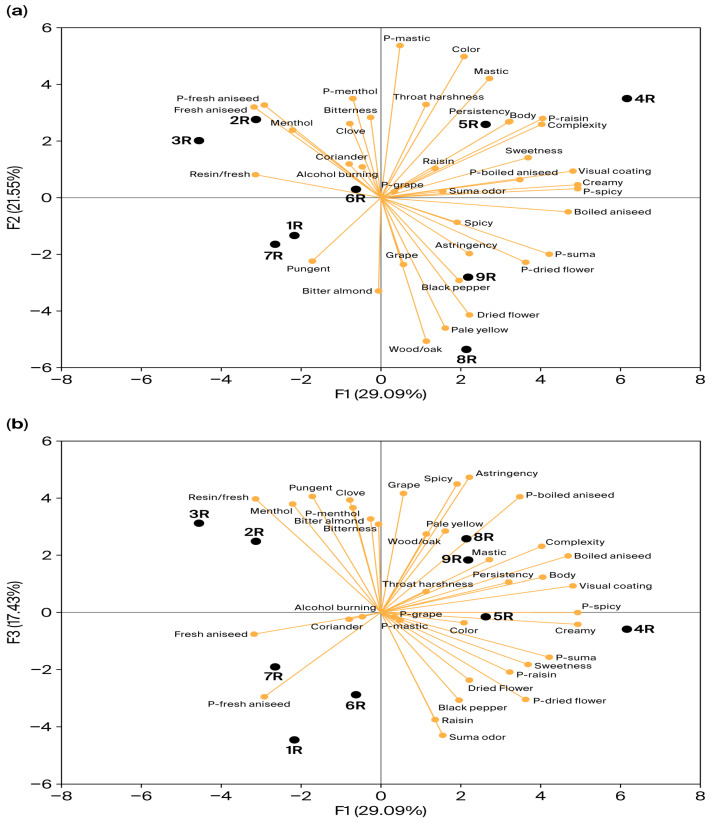
Principal component analysis of descriptive analyses of rakı. (**a**), PC1 and PC2 dimensions; (**b**), PC1 and PC3 dimensions; black points with codes 1R–9R represent rakı samples as active observations; orange vectors represent the sensory attributes as variables. P, on palate (retronasal aroma).

The sensory evaluation data are visualized using principal component analysis (PCA), as shown in Figure 2. PC1 explains 29.1% of the variance, PC2 represents 21.6% of the variance, PC3 accounts for 17.4% of the variance, and PC4 explains 11.8% of the variance, totaling up to 79.9% of the variance. PC4 and PC5 were excluded for the purpose of simplicity, as they had minimal impact on the outcome.

The components PC1, PC2, and PC3 divided the products into four distinct groups (Figure 2). Products 5R and 4R were grouped together and showed a positive correlation with attributes such as creamy, p-spicy, mastic, visual coating, body, raisin, complexity, persistency, and color. This clustering is directly attributed to their elevated trans-anethole concentrations (2.0–2.1 g/L) and high alcohol strengths (47.0–47.5% ABV, Table 1), which enhance the white color and provide a full-bodied mouthfeel. In these products, the high anethole content enhances both the aroma intensity and the whitening effect. The appearance of rakı is defined by the solubility of aniseed-derived anethole (typically >0.8 g/L) in its 40–50% ABV matrix [5]. Upon dilution with water, the decrease in ethanol concentration renders these essential oils insoluble, creating a rapid, bright white emulsion (whitening). The quality of this transition, along with the subsequent apparent visual coating on the glass, is directly correlated with the aniseed’s quality, quantity, and freshness [6]. Correspondingly, products 4R and 5R displayed a dominant visual coating and intense whiteness, aligning with their high-level anethole content, as shown in Table 1.

In contrast, products 3R and 2R formed another group, positively correlated with fresh aniseed, menthol, resin/fresh, bitterness, and clove attributes. This profile stems from the use of fresh grape Suma and, specifically for 2R, first-harvest aniseed (*Pimpinella anisum* L.). Aroma analysis revealed that these beverages were characterized by a high abundance of highly volatile monoterpenes that contribute to a fresh aroma. The PCA correlation matrix indicated that menthol attributes display a positive correlation with cloves (*r* = 0.82) and a negative correlation with dried flowers (*r* = −0.87). According to Darıcı et al. [6], a descriptive analysis of 18 rakı samples showed a significant relationship between the rakı categories (related to Suma source and aniseed level) and sensory attributes, particularly flavor intensities.

A third group, comprising products 1R, 6R, and 7R, is associated with pungent and bitter almond attributes. This pungency may be attributed to the partial reuse of head and tail cuts or a lower proportion of grape Suma (at least 65% of the total distillate) and the inclusion of agricultural ethanol, combined with relatively lower trans-anethole concentrations (1.2–1.3 g/L). This composition reduces the sensory masking effect, making the trigeminal sharpness of the ethanol more pronounced. Consistent with Darıcı et al. [6], the findings indicate that the addition of heads and tails or lower Suma proportions (65%) reduce aromatic diversity while enhancing pungent perceptions. Furthermore, the bitter almond aroma in these samples (notably 1R and 7R) is quantitatively linked to elevated p-anisaldehyde concentrations (up to 2747 µg/L, Table S2), as confirmed by our previous GC-O study [7].

Finally, products 8R and 9R were distinctly grouped by attributes of pale-yellow color, dried flower, wood/oak, and astringency. The attributes of pale-yellow color, astringency, and woody aroma distinctly differentiate these two products from the others. This outcome is expected, as products 9R and 8R have undergone treatment in short-term oak barrels.

Overall, the correlation matrix highlights that complexity and persistency are closely associated with mouthfeel attributes. Complexity showed positive correlations with mastic (*r* = 0.81), visual coating (*r* = 0.86), body (*r* = 0.86), creamy (*r* = 0.80), and p-spicy (*r* = 0.80). Similarly, persistency was positively correlated with color (*r* = 0.73), mastic (*r* = 0.72), body (*r* = 0.86), and throat harshness (*r* = 0.89). These findings indicate that aroma compounds from aniseed—particularly anethole, estragole, and γ-himachalene—contribute to mastic, menthol, fresh, and spicy odors [7], which also affect mouthfeel, especially body and creamy attributes. Consequently, the specific Suma source, aniseed harvest quality, and maturation techniques collectively shape the sensory characteristics of rakı, ranging from raisin, spicy, and mastic odors (5R, 4R) to fresh, resin, and menthol profiles (2R, 3R). Among the various aroma attributes identified in this study, the most prominent were fresh and boiled aniseed, menthol, mastic, resin/fresh, spicy, grape, black pepper, and dried flower. These results align with previous research that identified a varied sensory lexicon for rakı, including 78 attributes such as spicy, anise, sweet, resinous, fruity, dried fruit, floral, head and tail aroma, and white color [6]. In another sensory lexicon study for the anise-flavored spirit Ouzo, eight odor attributes were determined as anise, mastic, sweet, alcoholic, herbs, vanilla, menthol, and strong [45].

Associating the instrumental aroma data with the descriptive sensory profiles provides a comprehensive understanding of rakı. The chemical groupings identified in the aroma PCA (Figure 1) appear to be closely related to the sensory clusters from the DA (Figure 2). For instance, the oak-aged samples (8R and 9R), which were chemically characterized by high levels of volatile phenols and oxygenated monoterpenes (such as d-carvone), aligned with the sensory attributes of wood/oak, pale yellow, astringency, and dried flower. The volatile phenols are indicated to derive from oak lignin degradation during the barrel maturation process [9]. Conversely, products 4R and 5R, which contained the highest concentrations of phenylpropanoid derivatives (particularly trans-anethole) and sesquiterpenes (such as γ-himachalene), were profiled by visual coating, body, creamy, mastic, persistency, and complexity. As supported by a previous study on the sensory lexicon of rakı [6], the abundant presence of sesquiterpenes and aniseed-derived compounds like anethole and estragole appears to contribute not only to aroma intensity but also to the mouthfeel, trigeminal perception, and viscosity of the beverage. Similarly, products 2R and 3R, which were chemically distinguished by their high monoterpene and monoterpenoid alcohol content (such as d-limonene and linalool), tended to correspond with the fresh aniseed, menthol, and resin/fresh sensory descriptors. Limonene and linalool are well known for giving citrus, fresh, and floral/lavender odors to aniseed-based spirits and gins [8,12]. Additionally, the concentration of certain esters appeared to influence specific fruity perceptions. Notably, product 5R was associated with the raisin attribute, characterized by the highest concentration of ethyl hexanoate, an ester widely recognized for its fruity aroma [7,10].

### 3.3. Demographic Profile and Consumer Preference

Participants in the sensory analysis were primarily regular rakı consumers who consumed the beverage several times a month (at least three times) or several times a year (at least eight times). The panel, representing Millennials and Generation Z, included 47.0% within the 30–45 age range and 38.0% within the 18–29 age range. Regarding gender distribution, the overall panel consisted of 46.0% females and 54.0% males (Supplementary Table S4).

Regarding general consumption frequency, 42.0% of all participants reported consuming rakı several times a month, followed by 38.0% who consumed it several times a year. Age was a determining factor in consumption habits; drinking “several times a year” was more common in the 18–29 age group (*N* = 38, 42.1%) compared to “several times a month” (31.6%). In contrast, participants aged 30–45 (*N* = 47) reported more frequent consumption, with 44.7% drinking rakı several times a month (Supplementary Table S5). Similar to the increase in wine consumption among younger generations in traditional wine markets such as the US and Australia [30,46], a corresponding increase in rakı consumption was observed within this demographic. Moreover, this trend is also comparable to whiskey consumption. According to Hamilton et al. [47], a study of 520 US whiskey consumers who drink at least once per month found that 30% of respondents were female and 69.6% were male, and the most dominant groups were the 24–34 age range, representing 31.7%, and the 35–49 age range, constituting 41.0%.

On the other hand, consumers aged between 46 and 65 in this study (15.0%), primarily Generation X, were identified as traditional consumers. Eighty percent of these participants consumed rakı either multiple times a month or once a week. Thus, although younger generations show increasing interest in rakı, older consumers maintain a higher frequency of consumption, possibly influenced by economic factors, as income often tends to increase with age. According to Patwardhan et al. [48], the most influential factors that affect Indian consumers’ choice of whiskey are aging, price, and packaging. Their study highlighted that price is a critical aspect and one of the key factors influencing whiskey purchase decisions in India.

To analyze consumer preferences for rakı, an Agglomerative Hierarchical Clustering Analysis (AHC) was conducted, grouping consumers into two distinct clusters based on their liking scores (Table 2).

Cluster 1 (*N* = 55) consisted of consumers who gave significantly higher liking scores compared to Cluster 2 (*N* = 45). The differences in preference were significant (*p* < 0.05) for all samples, with the exception of product 6R, where no significant difference was observed. The similar scores for 6R from frequent consumers in Cluster 1 (6.32) and casual consumers in Cluster 2 (6.12) show that this sample represents a sensory balance that combines aromatic intensity with accessibility. As shown in Table 2, Cluster 1 had average liking scores ranging between 6.32 and 7.0, while Cluster 2 had average liking scores between 4.67 and 6.12. Furthermore, the differences between the clusters were most pronounced for samples 5R and 8R. Cluster 1 appreciated the pronounced alcohol influence of 5R and the aromatic complexity of oak-aged 8R, whereas Cluster 2 gave considerably lower ratings to these samples.

When evaluating the clusters based on demographic profiles, the association between the clusters and gender approached statistical significance (*p* = 0.08). Cluster 1 had a higher proportion of male participants (61.8%) compared to Cluster 2 (44.4%). These results are partially consistent with the findings of Alencar et al. [30], who reported significant gender differences in the preference for Syrah wines treated with oak chips, noting that a male-dominant group disliked the samples while a female-dominant group appreciated them. However, in the present study, age and consumption frequency were the more definitive and statistically significant key factors of differentiation between the clusters (Table 2). Cluster 1 was characterized by consumers aged 30–45 (56.4%) who consumed rakı several times a month. In contrast, Cluster 2 represented a younger demographic (18–29 years; 57.8%) with a lower or rare consumption frequency. This suggests that frequency of consumption and age are more effective than gender in determining rakı preferences.

Rakı is a complex distilled beverage with various aroma compounds and sensory attributes [6,7], sharing similarities with whiskey in terms of complexity. As Patwardhan et al. [48] revealed for Indian whiskey, factors like aging, packaging, and price are significant drivers of consumer preferences. Similarly, beer preferences are known to be influenced by attributes such as flavor, age, brand image, and price [49]. Considering these similarities across different beverages, these factors, particularly the relationship between perceived quality and economic factors, should be investigated for rakı in future studies to better understand consumer behavior. 

### 3.4. Consumer Sensory Profiles by Check-All-That-Apply

The Check-All-That-Apply (CATA) method, widely utilized in sensory and consumer science, facilitates market research of food products and the characterization of their sensory properties [18]. This method is designed to allow participating consumers to select sensory attributes from a provided list based on their perceptions [50].

In the present study, the sensory descriptors were initially identified and defined by a descriptive analysis (DA) panel to establish the main sensory character of rakı. For the consumer-based CATA question, these main attributes were further expanded and refined into a more practical and comprehensive list to better capture consumer perceptions. Therefore, the DA results served as an essential basis for the CATA evaluation of rakı, particularly since previous research is limited. These descriptors, including appearance (greyish white, white, intense bright white, and slightly yellow), aroma, and taste/flavor properties, were used as the primary descriptors for the products. Initially, the counts of checked attributes from the CATA question across all consumers were determined (Supplementary Table S6). Accordingly, a contingency table reflecting the proportions of selection was generated, as shown in Appendix A.3, expressing the frequency with which each attribute was selected by 100 panelists across nine rakı samples. In the present study, a total of 43 CATA attributes selected by the assessors were used to characterize the nine rakı samples. Attributes with a selection frequency of less than 10% were identified as sparse attributes (eight attributes) and were excluded from further analysis to avoid overemphasizing rare attributes.

As detailed in Supplementary Table S6, attributes such as fresh aniseed (66%) and persistence (62%) exhibited the highest selection frequencies, reflecting the main sensory characteristics shared across all samples. According to Appendix A.3, significant differences (*p* < 0.05) were observed in all appearance categories (greyish white, white, intense bright white, and slightly yellow) and glass coating levels (low, medium, and high coating). Among the aroma and taste/flavor terms, cooked/boiled aniseed, raisin, aromatic, harsh, and smooth were identified as the key attributes that differentiated the samples.

**Figure 3:**
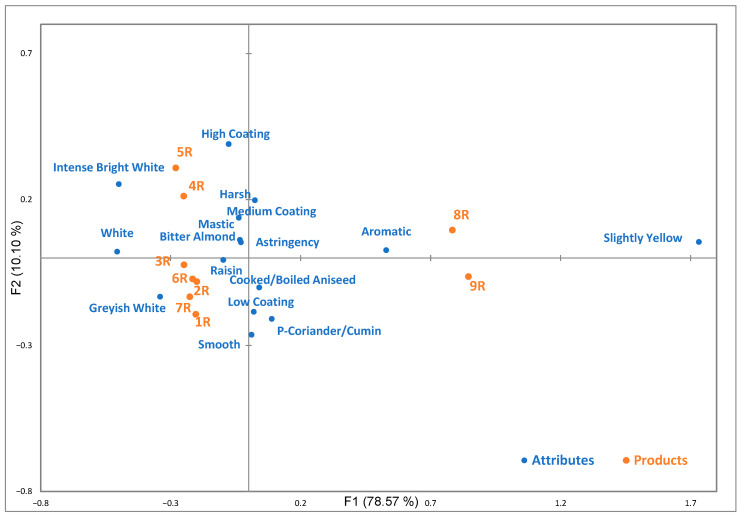
Symmetry biplot of corresponding analysis of CATA data for significant sensory attributes and rakı products. Attributes that did not differ significantly across rakı samples (*p* > 0.20) according to Cochran’s Q test were excluded.

Subsequently, correspondence analysis (CA) was performed to visualize the sensory associations between the rakı samples and their respective descriptors, as shown in Figure 3. The symmetric plot of CA using the chi-square distance explains 88.67% of total inertia on the first two dimensions. Figure 3 shows that the first dimension (F1) distinctly separates products 8R and 9R from the other products. These two samples are positioned on the positive side of F1 and are strongly characterized by the attributes slightly yellow (*p* < 0.0001) and aromatic (*p* < 0.0001).

Products 8R and 9R were distinguished from the main cluster due to their treatment in short-term oak barrels. This distinct separation is consistent with the present DA and a previous DA study where oak-aged rakı was found to group separately from other products; furthermore, they were defined primarily by woody and pale-yellow attributes [6]. The specific aging process contributed to the aromatic attributes and slightly yellow appearance in the CATA study. The association of the aromatic attribute specifically with oak-aged products suggests that consumers categorize oak-derived aroma compounds under an aromatic group. Even short-term oak aging may be sufficient to transform the traditional aniseed-based sensory profile into a more complex aromatic profile that is easily distinguished by consumers.

The aromatic grouping suggests a consumer tendency to use general terms instead of complex aging-related descriptors such as oak, vanilla, and sherry. Corsi et al. [51] observed that wine consumers in China used more generic terms compared to experts. Moreover, according to Hamilton et al. [47], less familiar and non-food-related terms might be ignored by consumers. They indicated that whiskey consumers prefer more familiar and understandable flavor descriptors instead of the specific terms used by professionals. Consequently, the CA successfully captured these characteristics, clearly differentiating the samples treated in short-term oak barrels from the classic rakı samples (1R–7R), which remained clustered around attributes such as white and raisin.

The second dimension (F2) further separates this group based on aroma, flavor, and mouthfeel. Samples 4R and 5R are more closely related to high glass coating (*p* = 0.00), intense bright white, mastic, bitter almond, and harsh (*p* = 0.04) attributes. This positioning can be explained by their higher anethole and alcohol content relative to the other products; the glass coating, intense white, bitter almond, and mastic attributes were likely influenced by the anethole concentration, while the harsh attribute represents the elevated alcohol content. Previous research [6] demonstrated that premium rakı categories with a high alcohol content were related to burning, throat burning, and harshness, which is considered a favorable characteristic of rakı. The finding of harsh sensations at higher alcohol levels is similar to studies on whiskey which demonstrated how ethanol levels affected by dilution can significantly change the perception of sensory profiles [9]. In contrast, the other samples (1R, 2R, 3R, 6R, and 7R) are associated with cooked/boiled aniseed, smooth, greyish white, and low glass coating attributes, defining a separate sensory cluster. Darıcı et al. [7] reported that as the number of distillations increased, the total amount of volatile compounds decreased, but this result made the perception of the desired aroma active compounds (such as anethole, estragole, linalool) more distinct and stronger. The present research indicates that the sensory differences identified by the CATA approach are not only due to aroma intensity, but also due to differences caused by the number of distillations.

Differences in the characteristics of aniseed, whether fresh or cooked, indicate that fresh aniseed attributes (*p* = 0.65) did not exhibit significant differences across the products. In contrast, cooked/boiled aniseed (*p* = 0.02) demonstrated significant differences. The findings indicate that while fresh aniseed is defined as a primary descriptor for all samples, the main sensory differences occur through the cooked/boiled aniseed odor. This characteristic may reflect differences in the distillation process, such as the inclusion of head and tail fractions, or highlight differences in the quality of the aniseed used, particularly in distinguishing product 1R from the others. Boiled aniseed and head and tail aromas have previously been reported as key indicators for lower-end rakı categories, often resulting from the addition of head and tail cuts and excessive recycling during pot still distillation [6]. The influence of these distillation fractions on sensory quality is also supported by recent research. Xiang et al. [52] observed that the addition of a minimal quantity of head distillate into the heart distillate influences the overall aroma and flavors of wine spirits. During distillation, adding the “head” cut in controlled quantities (about 0.38% *v*/*v*) into the heart cut could improve the fruity characteristic of the distillate; however, when it surpasses 0.41%, the “solvent/chemical” odor becomes predominant. Moreover, the determination of distinct attributes showed the capacity of the CATA method to identify quality or process-related descriptors or defects from the distillation process. Similar observations were reported in a sensory wheel study of grape marc spirits, where CATA and RATA were effectively used to refine a comprehensive list of descriptors and identify the key descriptors [53].

Although astringency (*p* = 0.09) is typically expected to be more pronounced in barrel-aged samples due to the presence of oak-derived tannins, it was perceived at moderate levels across all samples without a clear dominance in the oak-treated products. This finding suggests that consumers may not have clearly distinguished astringency from the burning sensation associated with high alcohol content. For untrained participants, trigeminal sensations such as sharp, pungent, and alcohol burning might be perceived as overlapping with tactile sensations like astringency in distilled products. Nolden and Hayes [54] observed in their study on the sensory perception of ethanol that untrained participants had difficulty distinguishing trigeminal sensations (burning/tingling). Additionally, Ickes and Cadwallader [55] observed in their study that high-alcohol rum samples (undiluted) received higher “astringency” scores. Moreover, McDonnell et al. [56] observed that dynamic sensory learning through successive DA sessions can stabilize these perceptions, as untrained assessors may initially lack the discrimination required to distinguish between complex, overlapping attributes. In particular, throughout the three DA sessions, the panelists initially found it difficult to discriminate between grappa samples associated with woody, alcohol, grain, musty, rubbery, pine, and musky attributes. Significant discrimination between the grappa samples was only achieved by the final DA. Overall, the integrated approach of DA-verified attributes and consumer-based CATA profiling effectively mapped the sensory characterization of rakı. The findings underscore that while aniseed remains the main descriptor, differences in alcohol or anethole content and oak treatment significantly shift consumer perception, allowing for distinct product positioning in the market.

In conclusion, comparing the DA and CATA results clearly shows the necessity of presenting both datasets for the rakı products. Both methods successfully distinguished the oak-aged samples (8R and 9R) from classic-style rakı, proving that CATA is a reliable and rapid tool. However, the differences between the two methods are equally important because they reveal a clear gap between expert and consumer terminologies. While the trained DA panel easily identified complex mouthfeel attributes like “astringency”, “body”, and “creamy”, naive consumers struggled with these terms. Instead of understanding these complex words, consumers often relied more on visual cues (such as glass coating) or basic burning sensations. Therefore, presenting both DA and CATA is essential to identifying not only the sensory profile of rakı but also how naive consumers actually perceive and describe it.

### 3.5. Sensory Impact on Consumer Preference

**Figure 4:**
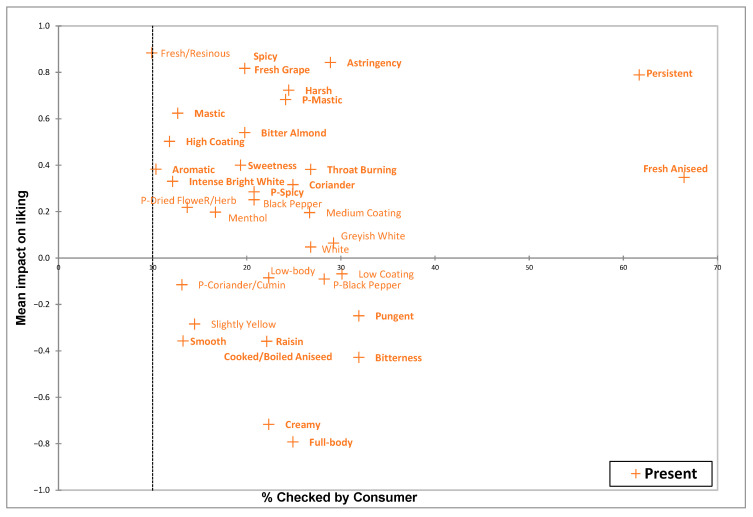
The impact of each CATA descriptor on average liking. The x-axis represents the percentage of consumers who checked the attribute while the y-axis represents the change in liking. Attributes highlighted in bold significantly impact liking (*p* < 0.05). The dotted line represents the 10% threshold for consumer-checked percentages.

To identify the key attributes contributing to consumer preference, a penalty analysis was conducted by evaluating CATA descriptors in combination with overall liking scores (Figure 4). This analysis highlights how specific sensory characteristics directly impact the average preference for rakı samples. To determine the significance of these characteristics, two-sample *t*-tests were performed to compare the differences in average liking scores when an attribute was checked (Present) versus when it was unchecked (Absent) by consumers. This approach allowed for the determination of the CATA attributes that have a significant positive or negative impact on consumer preferences across all products.

Figure 4 displays the mean impact on liking versus the percentage of consumers who selected each attribute. The attributes positioned above the x-axis represent the primary positive influencers of liking, indicating that their presence significantly enhances a product’s liking score. Attributes such as fresh aniseed, persistence, spicy, fresh grape, harsh, astringency, p-mastic, mastic, bitter almond, high glass coating, sweetness, aromatics, throat burning, intense bright white, coriander, and p-spicy are in the upper quadrants of Figure 4, showing a significant positive impact on consumer preference. Conversely, the attributes located below the x-axis indicate sensory descriptors that decrease the average preference for a product. Descriptors such as pungent, smooth, raisin, cooked/boiled aniseed, bitterness, creamy, and full body are positioned on the negative side of the y-axis, reflecting the significant negative impact of these attributes that decrease the overall liking score.

The penalty analysis emphasizes the significant impact of production processes on consumer preferences. Attributes related to raw component quality, such as fresh aniseed and fresh grape, significantly improve liking. In contrast, attributes associated with processing characteristics, specifically cooked or boiled aniseed, were observed to negatively impact consumer preference. This distinction emphasizes that maintaining fresh odors is a crucial factor in attracting consumers.

Moreover, a notable inconsistency was observed in the perception of mouthfeel characteristics. Consumers distinctly preferred characteristics such as high glass coating, mastic, bitter almond, and menthol, which are primarily sourced from aniseed; however, consumers penalized the mouthfeel sensory characteristics, including creamy and full body. Consumer preference for high glass coating, mastic, and bitter almond attributes was identified as a key driver. According to Darıcı et al. [6], the premium rakı category shows a positive correlation with attributes such as aniseed, mastic, spiciness, menthol, persistency, mouth coating/creaminess, body, and sweetness. An elevation in aniseed-derived compounds generally increases the viscous and coating mouthfeel characteristic of the beverage, thus resulting in a full-body and creamy mouthfeel.

Nevertheless, while the consumers similarly preferred persistence and harsh characteristics, it is suggested that they may not have understood the complex terms “creamy” and “full body”. Consumers may perceive these descriptors as a lack of the expected persistence that defines the traditional beverage’s properties. Consequently, they may misinterpret these mouthfeel attributes as signs of a low-impact product, showing a clear preference for trigeminal sensations and overall beverage persistence.

**Figure 5:**
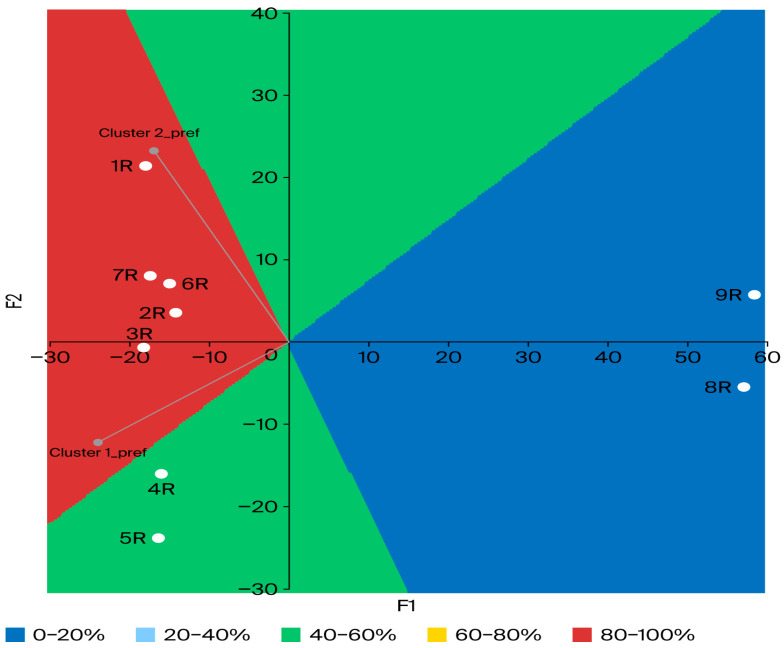
External preference mapping with contour graph. Created with CATA data and liking clusters grouped with AHC. Cluster 1_pref: average liking of Cluster 1; Cluster 2_pref: average liking of Cluster 2. Vectors are cluster average liking and points are rakı products. Colored regions indicate the percentage of consumers scoring average or above-average liking.

To further understand the differences in consumer preferences, external preference mapping (PREFMAP) was conducted, as presented in Figure 5. The resulting relationship between consumer clusters based on liking and rakı products is visualized using a contour plot.

The distance biplot was constructed using a covariance matrix, explaining 85.59% of the total variance on the first two dimensions. In this model, the clusters formed based on consumer liking scores (see Table 2) are represented as vectors, while the rakı products are displayed as individual points. Consequently, the preference relationship between the cluster liking (vectors) and the rakı products (points) is mapped in Figure 5. The contour plot displays colored regions corresponding to various levels of preference, indicating the percentage of consumers scoring average liking or above the average liking. In Figure 5, regions characterized by cool colors (blue) indicate a low percentage of preference, whereas regions with warm colors (red) signify a high preference percentage. Consequently, the red-colored zone in Figure 5 represents the potential “ideal product region”. This red region indicates where 80–100% of consumers are expected to score the product at or above the average preference level.

Products that are close together exhibit similar sensory properties and were allocated to specific preference zones on the map. Products 3R, 2R, 6R, 7R, and 1R are located in the red “ideal zone”, where 80–100% of consumers gave above-average liking scores. Products 4R and 5R are positioned in the green zone, suggesting intermediate acceptability (40–60%), while products 9R and 8R are found in the blue zone, showing that they were the least favored. In terms of cluster preference, Cluster 1 had a particularly positive response to the aromatic complexity derived from short-term oak treatment, whereas Cluster 2, comprising younger and less frequent consumers, gave notably lower scores of 4.67 and 5.04, respectively (see Table 2). This indicates that the slightly yellow appearance and oak-derived aromatic aromas represent a sensory deviation from the traditional “white” rakı identity that this category expects. Sensory profiles that fail to meet category-specific sensory expectations or that elicit a mismatch between consumer expectations and experiences may not be preferred, even if they possess an aromatically complex and rich profile [57,58].

According to Hamilton et al. [47], professional reviewers often describe expensive whiskies using specific terms like sultana, oak, leather, and tobacco; in contrast, they reported that American consumers are more willing to purchase whiskies with more familiar and sweet flavor characteristics, such as chocolate and caramel. They noted that these terms do not appeal to whiskey consumers as much as they do to experts. Moreover, premium quality products 4R and 5R in the present research fell into the intermediate preference category, although they had a more pronounced association with Cluster 1, which includes more frequent and experienced consumers. This suggests that the high alcohol content and anethole concentrations of these products, which contribute to their mastic, harsh, and high glass coating sensory profiles, are preferred by experienced consumers. Villanueva et al. [27] determined that young consumers (18–34 years old) preferred lower-priced wines over those ranked highest by experts and exhibited the lowest frequency of wine consumption. Conversely, it was observed that the middle-aged demographic (25–44 years) preferred wines that aligned with the quality characteristics of the expert panel and exhibited the highest frequency of consumption.

Cluster 2, consisting of younger and more casual consumers, tended to prefer products without intense trigeminal sensations such as burning and harshness. Nolden and Hayes [54] observed that the perceived intensity of the sensory effects of ethanol (especially bitterness, burning, and harshness) is related to an individual’s drinking frequency; for inexperienced (naive) individuals who do not consume alcohol frequently, these characteristics are the key criteria affecting preference and consumption. On the other hand, experienced consumers are likely more tolerant of these effects or interpret them differently. Furthermore, Patwardhan et al. [48] determined that aging, price, packaging, and aftertaste/aroma influence consumer preferences in the whiskey industry. They also highlighted that competitive pricing, innovative packaging, and aroma are essential strategic instruments for attracting and retaining young consumers. Additionally, the DA and CATA results showed a similar distribution of the products in PCA and CA. However, due to the presence of inexperienced (naive) consumers, some mouthfeel and trigeminal attributes such as burning, astringency, creamy, and body did not align with the direction given by trained panelists. These results indicate that while consumers are sensitive to major aromatic variations, they struggle to discriminate between complex tactile and trigeminal sensations. Similar to these findings, Alencar et al. [31] observed consistency between DA (trained panelists) and CATA (consumer) results for Syrah wines aged with American and French oak chips. Consumers in that wine study were able to perceive differences in spice, vanilla, and woody aromas through CATA, and their preferences were more influenced by the type of oak chip than aging duration.

## 4. Conclusions

This study investigated the sensory profile of rakı by integrating trained panel descriptive analysis with consumer-based CATA, illustrating that CATA is a highly effective tool for characterizing and distinguishing complex distilled beverages. This research represents a novel approach by applying the advanced SBSE-GC-MS technique to rakı for the first time, while pioneering the integration of consumer-based CATA with consumer profiling and preference mapping. The findings indicate “fresh aniseed” and “persistency” as the key sensory characteristics. Product differentiation and consumer preference are primarily influenced by processing variables, including distillation cuts—particularly the removal of “boiled/cooked” off-flavors—as well as the levels of anethole and alcohol concentrations. Instrumental analysis supported the research by identifying 81 aroma compounds, establishing that while trans-anethole and estragole constitute the primary profile, the secondary aroma composition displays considerable variation; specifically, monoterpenes dominate in certain products (1R, 2R, and 3R), whereas sesquiterpenes are predominant in others (such as 4R, 5R, and 6R). These differences, along with specific esters and volatile phenols, clearly differentiate the products based on their Suma origin/ratio, distillation program, and maturation process. A significant observation is the discrepancy between sensory descriptors and consumer terminology; consumers found conceptual mouthfeel descriptors such as “astringency”, “body”, and “creamy” challenging, instead favoring visual indicators such as “glass coating” and “whitening” to characterize the products.

Consumer profiling identified two distinct clusters, highlighting a pronounced division between experienced traditionalists and the younger demographic. Cluster 1, consisting of older and more frequent consumers, preferred complex, high-alcohol beverages with a high anethole content and displayed tolerance for trigeminal “harshness”. On the other hand, Cluster 2, comprising younger and more casual drinkers, favored smoother, traditional “white” appearance profiles and responded negatively to the visual deviation of the slightly yellow, barrel-aged samples. Therefore, producers must prioritize the removal of “boiled” defects for overall acceptability while implementing a targeted marketing and positioning strategy: emphasizing “visual coating” and complexity for premium markets and providing smooth, visually conventional profiles to attract the growing younger demographic. Future research should incorporate external factors, such as pricing, packaging, and brand image, to understand how these variables influence the purchase decisions of the younger demographic. Furthermore, future projects could concentrate on developing a consumer-oriented sensory lexicon to bridge the gap between trained assessor analysis and consumer perceptions. Additionally, while the nine commercial samples evaluated in this study represent the major quality categories and highest market volumes, future research could expand the sample size to include a wider range of rakı products to further validate these findings across the entire market.

## Figures and Tables

**Table 1:** Main properties of rakı samples used in the present research.

Product Code	Suma Source ^δ^	Category ^γ^	Proportion of Suma	Head and Tail ^ζ^	Alcohol Strength (%ABV)	Sugar Content (g/L)	Trans- Anethole Content (g/L)	Application
1R	Fresh grape and/or raisin	Mass	≥65% ^ε^	√	45.0	4.5	1.3	Classic style, multiple distillation cycles.
2R	Fresh grape and/or raisin	Mass	≥65%	√	45.0	4.5	1.4	Aniseed from the first harvest of the year is used in production.
3R	Fresh grape	Mass	≥65%	-	45.0	5.0	1.4	100% fresh grape Suma.
4R	Fresh grape	Premium	100%	-	47.5	3.0	2.1	Triple distillation, 100% fresh grape distillate Suma. Produced only in single pot still.
5R	Raisin	Premium	≥65%	-	47.0	2.0	2.0	Triple distillation, 100% raisin Suma.
6R	Fresh grape	Premium	≥65%	-	45.2	2.0	1.3	Triple distillation, 100% fresh grape Suma.
7R	Fresh grape	Premium	≥65%	-	45.0	nd	1.2	Triple distillation, 100% fresh grape Suma.
8R	Fresh grape	Mass	100%	√	45.0	5.0	1.5	100% fresh grape Suma. Maturing for approximately 2–3 months in oak barrels.
9R	Fresh grape	Mass	100%	-	45.0	nd	1.4	100% fresh grape Suma. Maturing got approximately 2–3 months in oak barrels.

^γ^ category information is taken from each producer; ^δ^ Suma, grape or raisin distillate; ^ε^ at least 65% of the total distillate is Suma; ^ζ^ samples with √ have partially reused head and tail cuts in pot still distillations. nd, not detected; all chemical analyses (alcoholic strength, sugar and anethol content) were performed with three replicates and the standard deviation from the mean was under 5%.

**Table 2:** Demographic differences in liking of Rakı between two consumer clusters identified in AHC.

		Cluster 1 *N*: 55	Cluster 2 *N*: 45		
**Liking**		**Mean values of clusters**		**F*p***	
1R		7.00 ^a^	5.31 ^b^	<0.0001	
2R		6.90 ^a^	5.35 ^b^	<0.0001	
3R		7.11 ^a^	5.22 ^b^	<0.0001	
4R		6.99 ^a^	5.70 ^b^	0.0001	
5R		6.74 ^a^	4.76 ^b^	<0.0001	
6R		6.32 ^a^	6.12 ^a^	0.57	
7R		6.83 ^a^	5.14 ^b^	<0.0001	
8R		6.80 ^a^	4.67 ^b^	<0.0001	
9R		6.53 ^a^	5.04 ^b^	<0.0001	
	**Total F (%)**	**Cluster F (%)**		**χ^2^*p***	**FET*p***
**Gender**					
Female	46.0%	38.2%	55.6%	0.080	0.11
Male	54.0%	61.8%	44.4%		0.11
**Age**					
18–29	38.0%	21.8%	57.8%	0.010	0.0004
30–45	47.0%	56.4%	35.6%		0.05
46–65	15.0%	21.8%	6.7%		0.05
**Consumption**					
Once a week	10.0%	9.1%	11.1%	0.030	0.75
Several times a month	42.0%	52.7%	28.9%		0.02
Several times a year	38.0%	34.5%	42.2%		0.54
Rarely	10.0%	3.6%	17.8%		0.04

AHC, Agglomerative Hierarchical Clustering Analysis. *N*, the number of consumers in each cluster. Cluster F (%) indicates the column (cluster) percentage of frequency. F*p* refers to the *p*-values obtained from two-way analysis of variance (ANOVA). Different superscripts (^a–b^) within the same row indicate pairwise comparisons among clusters based on Tukey’s HSD test at the *p* < 0.05 level. χ^2^ indicates *p*-values from chi-square analysis, and FET*p* refers to Fisher’s exact test *p*-values calculated for the within-cluster comparison.

## Data Availability

The original contributions presented in this study are included in the article/Supplementary Materials. Further inquiries can be directed to the corresponding author.

## Supplementary Materials

The following supporting information can be downloaded at https://www.mdpi.com/article/10.3390/foods15081321/s1, Table S1: The descriptors used in the CATA form and their corresponding reference list; Table S2: Aroma composition of rakı products; Table S3: Attributes’ mean scores for each rakı sample given by trained assessors in descriptive analysis; Table S4: Demographic profiles of participants; Table S5: Distribution of rakı consumption frequency by age group; Table S6: Counts of checked attributes by 100 panelists for CATA.

## Abbreviations

ABV	Alcohol by Volume
AHC	Agglomerative Hierarchical Clustering Analysis
ANOVA	Analysis of Variance
CA	Correspondence Analysis
CATA	Check-All-That-Apply
DA	Descriptive Analysis
DoE	Design of Experiments
FET	Fisher’s Exact Test
GC-FID/MS	Gas Chromatography–Flame Ionization Detector/Mass Spectrometry
GDA	Generic Descriptive Analysis
PCA	Principal Component Analysis
PDMS	Polydimethylsiloxane
PDO	Protected Designation of Origin
PrefMap	Preference Mapping Analysis
SBSE	Stir Bar Sorptive Extraction
TDU	Thermal Desorption Unit
UPC	Universal Peltier Cooling

## Appendix A

### Appendix A.1. Aroma Compounds and Their Corresponding Codes Used in PCA

A1: 3-Methyl-1-butanol + 2-Methyl-1-butanol; Ad1: 2-Diethoxymethyl-3-methyl-butyraldehyde; Ad2: Nonanal; Ad3: Decanal; Ak1: 2,3-dimethyl-2-Butene; Sa1: Spathulenol; Sa2: Isospathulenol; E1: Ethyl butanoate; E2: Ethyl-2-methyl butanoate; E3: Isoamyl acetate; E4: 2-Methylbutyl acetate; E5: Ethyl pentanoate; E6: Ethyl hexanoate; E7: Ethyl heptanoate; E8: Ethyl octanoate; E9: Ethyl decanoate; E10: Ethyl tetradecanoate; E11: Ethyl dodecanoate; F1: 2-pentyl-Furan; M1: α-Pinene; M2: β-Pinene; M3: α-Phellandrene; M4: δ-3-Carene; M5: p-Cymene; M6: D-Limonene; M7: trans-beta-Ocimene; M8: α-Terpinene; M9: Geijerene; Ma1: Linalool; Mo1: 2-methoxy-p-Cymene; Mo2: Fenchone; Mo3: Dihydrocarvone; Mo4: p-Cuminic aldehyde; Mo5: D-Carvone; Mo6: Geranyl butanoate; Pp1: 1-Methoxy-4-[(E)-2-methoxyethenyl]benzene; Pp2: cis-Anethole; Pp3: p-Anisaldehyde; Pp4: Chavicol; Pp5: 1-Methoxy-4-(1-methylpropyl)-benzene; Pp6: 3,5-Dimethyl-2-(1-phenylethyl)phenol; Pp7: 4-Methoxyphenyl-2-propanone; Pp8: 2-Allyl-1,4-dimethoxybenzene; Pp9: o-Methyleugenol; Pp10: 4-Methoxyphenyl-1-propanone; Pp11: 2-Phenyl-2-pentene; Pp12: Dill apiol; Pp13: 2-(1-E-propenyl)-4-methoxyphenyl 2-methylbutanoate; Pp14: p-Vinylanisole; Pp15: p-Methoxycinnamaldehyde; Sa1: Cedran-8-ol; Sa2: cis-Farnesol; S1: δ-Elemene; S2: α-Longipinene; S3: α-Ylangene; S4: β-Elemene; S5: β-Ylangene; S6: β-Gurjunene; S7: γ-Elemene; S8: α-Himachalene; S9: cis-β-Farnesene; S10: 8,9-dehydro-Neoisolongifolene; S11: γ-Himachalene; S12: Germacrene D; S13: α-Elemene; S14: ar-Curcumene; S15: α-Zingiberene; S16: β-Himachalene; S17: δ-Cadinene; S18: β-Bisabolene; S19: β-Sesquiphellandrene; S20: γ-Dehydro-ar-himachalene; S21: α-Calacorene; S22: β-Vatirenene; S23: α-Cuprene; Vf1: 2,6-Dimethyl-phenol; Vf2: 2-Hydroxy-4-methoxyacetophenone; Vf3: 2-(1-hydroxypropyl)-4-methyl-phenol.

### Appendix A.2. A List of CATA Questions

**Appearance (by Looking)**	**Aroma (by Smelling)**	**Taste/Flavor (by Tasting)**
Greyish White	Pungent	Burning
White	Fresh Aniseed	Sweetness
Intense Bright White	Boiled/Cooked Anise ed	Bitterness
Slightly yellow	Fresh Grape	Astringency
Low Coating (glass)	Raisin	Full-body
Medium Coating (glass)	Spicy	Light-body (Watery)
High Coating (glass)	Mastic	Harsh/Hard to drink
	Menthol	Smooth/easy to drink
	Fresh/Resinous	Creamy
	Black Pepper	Persistent
	Coriander	Throat Burning
	Dried Flower/Herb	Spicy
	Sweet Odor	Black Pepper
	Woody	Dried Flower/Herb
	Bitter Almond	Fresh Aniseed
	Aromatic	Boiled/Cooked Aniseed
		Mastic
		Coriander/Cumin
		Fresh Grape
		Raisin

### Appendix A.3. A Contingency Table of the Proportions of Selection by 100 Panelists Across All Samples for Attributes of the CATA

**Attributes ^γ^**	**1R**	**2R**	**3R**	**4R**	**5R**	**6R**	**7R**	**8R**	**9R**	**^β^ *p***
Greyish White	0.380 (cd)	0.420 (d)	0.390 (cd)	0.310 (bcd)	0.230 (abc)	0.360 (cd)	0.340 (cd)	0.060 (a)	0.140 (ab)	**<0.0001**
White	0.390 (b)	0.260 (b)	0.330 (b)	0.330 (b)	0.380 (b)	0.310 (b)	0.380 (b)	0 (a)	0.030 (a)	**<0.0001**
Intense Bright White	0.140 (ab)	0.130 (ab)	0.130 (ab)	0.190 (b)	0.220 (b)	0.160 (b)	0.100 (ab)	0.010 (a)	0.010 (a)	**<0.0001**
Slightly yellow	0.020 (a)	0.020 (a)	0 (a)	0.010 (a)	0.010 (a)	0.020 (a)	0 (a)	0.570 (b)	0.650 (b)	**<0.0001**
Low Coating	0.420 (c)	0.260 (abc)	0.280 (abc)	0.230 (ab)	0.180 (a)	0.350 (abc)	0.380 (bc)	0.260 (abc)	0.350 (abc)	**0.00**
Medium Coating	0.230 (ab)	0.330 (ab)	0.370 (b)	0.270 (ab)	0.330 (ab)	0.200 (ab)	0.170 (a)	0.260 (ab)	0.240 (ab)	**0.02**
High Coating	0.070 (ab)	0.050 (a)	0.090 (abc)	0.200 (c)	0.190 (bc)	0.150 (abc)	0.100 (abc)	0.130 (abc)	0.080 (abc)	**0.00**
Pungent	0.350 (a)	0.280 (a)	0.330 (a)	0.320 (a)	0.360 (a)	0.310 (a)	0.340 (a)	0.330 (a)	0.250 (a)	0.74
Fresh Aniseed	0.650 (a)	0.660 (a)	0.640 (a)	0.740 (a)	0.660 (a)	0.690 (a)	0.680 (a)	0.600 (a)	0.660 (a)	0.65
Cooked/Boiled Aniseed	0.340 (b)	0.240 (ab)	0.220 (ab)	0.150 (a)	0.170 (a)	0.220 (ab)	0.180 (ab)	0.280 (ab)	0.190 (ab)	**0.02**
Fresh Grape	0.230 (a)	0.200 (a)	0.270 (a)	0.190 (a)	0.180 (a)	0.180 (a)	0.190 (a)	0.130 (a)	0.210 (a)	0.36
Raisin	0.340 (b)	0.240 (ab)	0.220 (ab)	0.150 (a)	0.170 (a)	0.220 (ab)	0.180 (ab)	0.280 (ab)	0.190 (ab)	**0.02**
Spicy	0.230 (a)	0.200 (a)	0.270 (a)	0.190 (a)	0.180 (a)	0.180 (a)	0.190 (a)	0.130 (a)	0.210 (a)	0.36
Mastic	0.120 (a)	0.160 (a)	0.080 (a)	0.110 (a)	0.140 (a)	0.130 (a)	0.160 (a)	0.170 (a)	0.070 (a)	**0.18**
Menthol	0.160 (a)	0.130 (a)	0.200 (a)	0.210 (a)	0.180 (a)	0.120 (a)	0.160 (a)	0.180 (a)	0.160 (a)	0.61
Fresh/Resinous	0.090 (a)	0.070 (a)	0.130 (a)	0.090 (a)	0.130 (a)	0.120 (a)	0.070 (a)	0.080 (a)	0.110 (a)	0.49
Black Pepper	0.190 (a)	0.240 (a)	0.240 (a)	0.220 (a)	0.190 (a)	0.210 (a)	0.220 (a)	0.180 (a)	0.180 (a)	0.90
Woody	0.090 (a)	0.060 (a)	0.080 (a)	0.100 (a)	0.070 (a)	0.070 (a)	0.060 (a)	0.090 (a)	0.080 (a)	0.96
Bitter Almond	0.200 (a)	0.180 (a)	0.240 (a)	0.190 (a)	0.220 (a)	0.200 (a)	0.230 (a)	0.090 (a)	0.230 (a)	**0.14**
Aromatic	0.050 (a)	0.070 (a)	0.060 (a)	0.090 (ab)	0.050 (a)	0.090 (ab)	0.110 (ab)	0.210 (b)	0.200 (b)	**<0.0001**
Sweetness	0.260 (a)	0.230 (a)	0.170 (a)	0.200 (a)	0.160 (a)	0.140 (a)	0.220 (a)	0.210 (a)	0.150 (a)	0.23
Bitterness	0.260 (a)	0.340 (a)	0.320 (a)	0.350 (a)	0.310 (a)	0.310 (a)	0.370 (a)	0.330 (a)	0.280 (a)	0.80
Astringency	0.370 (b)	0.340 (ab)	0.290 (ab)	0.320 (ab)	0.300 (ab)	0.260 (ab)	0.180 (a)	0.300 (ab)	0.240 (ab)	**0.09**
Full-body	0.220 (a)	0.240 (a)	0.270 (a)	0.250 (a)	0.270 (a)	0.270 (a)	0.250 (a)	0.260 (a)	0.210 (a)	0.97
Low-body	0.240 (a)	0.170 (a)	0.190 (a)	0.200 (a)	0.210 (a)	0.270 (a)	0.220 (a)	0.240 (a)	0.270 (a)	0.63
Harsh	0.210 (a)	0.210 (a)	0.250 (a)	0.300 (a)	0.310 (a)	0.150 (a)	0.250 (a)	0.310 (a)	0.210 (a)	**0.04**
Smooth	0.160 (a)	0.140 (a)	0.130 (a)	0.100 (a)	0.050 (a)	0.170 (a)	0.180 (a)	0.080 (a)	0.180 (a)	**0.04**
Creamy	0.230 (a)	0.200 (a)	0.240 (a)	0.220 (a)	0.250 (a)	0.210 (a)	0.220 (a)	0.250 (a)	0.190 (a)	0.97
Persistent	0.550 (a)	0.620 (a)	0.590 (a)	0.620 (a)	0.590 (a)	0.700 (a)	0.600 (a)	0.640 (a)	0.640 (a)	0.62
Throat Burning	0.250 (a)	0.280 (a)	0.250 (a)	0.280 (a)	0.240 (a)	0.210 (a)	0.230 (a)	0.330 (a)	0.340 (a)	0.33
P-Spicy	0.190 (a)	0.210 (a)	0.200 (a)	0.200 (a)	0.200 (a)	0.200 (a)	0.240 (a)	0.240 (a)	0.190 (a)	0.98
P-Black Pepper	0.300 (a)	0.240 (a)	0.330 (a)	0.330 (a)	0.290 (a)	0.290 (a)	0.270 (a)	0.230 (a)	0.260 (a)	0.69
P-Dried Flower /Herb	0.160 (a)	0.120 (a)	0.140 (a)	0.150 (a)	0.160 (a)	0.120 (a)	0.120 (a)	0.120 (a)	0.140 (a)	0.95
P-Mastic	0.230 (a)	0.270 (a)	0.260 (a)	0.260 (a)	0.220 (a)	0.230 (a)	0.250 (a)	0.240 (a)	0.210 (a)	0.98
P-Coriander/Cumin	0.220 (b)	0.130 (ab)	0.130 (ab)	0.080 (a)	0.070 (a)	0.100 (ab)	0.150 (ab)	0.160 (ab)	0.140 (ab)	**0.02**
^γ^ Only attributes with a selection frequency of 10% or higher were included. ^β^ values represent Cochran’s Q test *p* values for each attribute. Values shown in bold color are significant at *p* < 0.2. Different letters in parentheses (a–d) in the same row indicate statistical differences according to Sheskin’s multiple comparison test at *p* < 0.05. Attributes starting with a P code are perceived in the palate (retronasal aroma).																				
