# Supplementary material for: Exploring The Sensory and Aroma Characteristics of Rakı Through Check-All-That-Apply and Consumer Preference Approaches
Source: Foods. 2026 Apr 10;15(8):1321. doi: 10.3390/foods15081321 (PMC13115056; doi:10.3390/foods15081321)
Supplement: Supplementary file 1 [file foods-15-01321-s001.zip › foods-4220035-supplementary.pdf]

# Supplementary Tables

Table S1. The descriptors used in the CATA form and their corresponding reference list

| Descriptors | Description/Explanation |
|---|---|
| Greyish White<br>White<br>Intense Bright White | Indicates the degree of whiteness. |
| Slightly Yellow | The degree of yellow color formed by aging in oak barrels. |
| Low Coating<br>Low Coating<br>Medium Coating | Appearance of oily and white streaks on the glass wall caused by anise oil (also expressed with terms like legs or tears). |
| Pungent | The smell and pungency of ethyl alcohol are perceived in the nose. |
| Fresh Aniseed | Sweet, refreshing, herbal, floral odors. |
| Boiled/Cooked Aniseed | Odor of cooked aniseed/seed. |
| Fresh Grape | Sweet, fruity, slightly acidic odors associated with grape aromas. |
| Raisin | Concentrated sweet, fruity, jammy odors are associated with raisin aromas. |
| Spicy | Spicy, slightly sweet and sharp odors are associated with aromas like black pepper, cloves, and fennel. |
| Mastic | Sweet, refreshing, elegant, resinous, gummy, turpentine-like odors. |
| Menthol | Refreshing, mint-related odors. |
| Fresh/Resinous | Resinous, refreshing odors like pine and fir. |
| Black Pepper | Spicy and sharp odors are associated with black pepper. |
| Coriander/Cumin | Spicy, soapy and sharp odors are associated with coriander and cumin seeds. |
| Dried Flower/Herb | Dry, soft, woody, dusty, floral odors are associated with dried flowers. |
| Sweetish Aroma | Confectionery and sweet odors related to aniseed. |
| Wood/Oak | Spicy, woody and dusty odors are associated with tree bark. |
| Bitter Almond | Spicy, bitter, sweet, woody and cherry-like odors are associated with bitter almond. |
| Aromatic | Caramelized sugar-like sweet aromas |
| Burning | The sensation of heat/stinging left by alcohol on the mouth and tongue. |
| Sweetness | Taste perception is perceived on the tongue, stimulated by sucrose. |
| Bitterness | Taste perception is perceived on the tongue, stimulated by quinine and caffeine. |
| Astringency | The sensation of dryness or roughness in the mouth caused by tannins. |
| Full-body | The sensation of fullness or weight that remains on the mouth/palate (combined effect of viscosity, flavor, and astringency). |
| Light-body (Watery) | Low sensation of fullness left on the mouth/palate. |
| Harsh/Hard to drink | Harsh, coarse sensation left by tannins and alcohol in the mouth. |
| Smooth/easy to drink | Soft, velvety sensation in the mouth. |
| Creamy | Intense and creamy/oily layer and coating sensation perceived on the tongue. |
| Persistent | The duration of the effect left on the senses after tasting (length). |
| Throat Burning | The sensation of heat/stinging caused by alcohol in the throat. |

## Supplementary Tables

Table S2. Aroma composition of rakı products (µg/L)

| Aroma Compounds | LRI | 1R | 2R | 3R | 4R | 5R | 6R | 7R | 8R | 9R | ID | *p* |
|---|---|---|---|---|---|---|---|---|---|---|---|---|
| ***Esters*** | | | | | | | | | | | | |
| Ethyl butanoate | 804 | 50 c ±9 | 43 c ±0 | 92 b ±0 | 32 cd ±1 | 123 a ±2 | 57 c ±15 | 36 cd ±10 | 51 c ±4 | 13 d ±5 | R,L,M | *<0.0001* |
| Ethyl-2-methyl butanoate | 848 | 425 ab ±20 | 423 ab ±9 | 479 a ±0 | 355 c ±16 | 177 d ±0 | 180 d ±23 | 158 d ±5 | 393 bc ±28 | 76 e ±18 | R,L,M | *<0.0001* |
| Isoamyl acetate | 877 | 302 bc ±26 | 265 c ±31 | 423 a ±0 | 206 c ±4 | 254 c ±10 | 196 c ±36 | 379 ab ±27 | 404 ab ±52 | 34 d ±6 | R,L,M | *<0.0001* |
| 2-Methylbutyl acetate | 881 | 39 a ±0 | nd | nd | nd | nd | nd | 55 a ±14 | nd | nd | R,L,M | *<0.0001* |
| Ethyl pentanoate | 905 | 60 ab ±2 | 58 ab ±1 | 78 a ±0 | 28 bc ±1 | 58 ab ±1 | 32 bc ±14 | nd | 66 ab ±27 | 8 c ±7 | R,L,M | *0.00* |
| Ethyl hexanoate | 1003 | 187 fg ±9 | 224 ef ±5 | 318 de ±0 | 434 cd ±12 | 1081 a ±49 | 458 c ±46 | 684 b ±24 | 375 cd ±9 | 71 g ±55 | R,L,M | *<0.0001* |
| Ethyl heptanoate | 1102 | nd | nd | nd | nd | 139 a ±9 | nd | nd | nd | nd | R,L,M | *<0.0001* |
| Ethyl octanoate | 1204 | 1069 b ±33 | 969 b ±281 | 1941 ab ±185 | 2352 ab ±452 | 646 b ±344 | 3162 ab ±2773 | 980 b ±52 | 3693 ab ±2 | 5220 a ±117 | R,L,M | *0.01* |
| Ethyl decanoate | 1400 | 201 de ±16 | 216 de ±46 | 809 c ±32 | 1348 a ±4 | 97 de ±66 | 341 d ±36 | 93 e ±27 | 1071 b ±71 | 149 de ±144 | R,L,M | *<0.0001* |
| Ethyl tetradecanoate | 1798 | nd | nd | nd | nd | nd | nd | 79 b ±3 | 99 a ±4 | nd | R,L,M | *<0.0001* |
| Ethyl dodecanoate | 1598 | 40 b ±0 | 40 b ±1 | 33 b ±0 | 45 b ±1 | 110 b ±7 | nd | 46 b ±2 | 122 b ±5 | 338 a ±124 | R,L,M | *0.00* |
| *Total* | | *2372 ab* | *2238 b* | *4172 ab* | *4799 ab* | *2686 ab* | *4426 ab* | *2511 ab* | *6273 a* | *5910 ab* | | *0.02* |
| ***Monoterpene hydrocarbons*** | | | | | | | | | | | | |
| α-Pinene | 928 | 395 a ±16 | 321 ab ±8 | 211 bc ±0 | 44 de ±2 | 80 de ±5 | 151 cd ±44 | 22 e ±5 | 211 bc ±78 | 34 de ±12 | R,L,M | *<0.0001* |
| β-Pinene | 969 | 191 b ±7 | 336 a ±6 | 146 bc ±0 | 32 e ±5 | 55 de ±1 | 99 cd ±18 | nd | 131 bc ±40 | nd | R,L,M | *<0.0001* |
| α-Phellandrene | 1001 | 112 c ±5 | 150 bc ±6 | 145 bc ±0 | nd | nd | 245 a ±32 | nd | 199 ab ±48 | nd | L,M | *<0.0001* |
| δ-3-Carene | 1006 | 38 bc ±20 | 22 bc ±2 | 30 bc ±0 | 0 c ±0 | 76 ab ±2 | 92 a ±29 | nd | 44 abc ±21 | 10 c ±4 | L,M | *0.00* |
| p-Cymene | 1021 | 519 ab ±25 | 635 a ±15 | 456 bc ±0 | 150 e ±17 | 281 d ±2 | 377 cd ±10 | 121 e ±8 | 488 bc ±77 | 60 e ±29 | L,M | *<0.0001* |
| D-Limonene | 1025 | 6936 a ±513 | 3504 bcd ±179 | 4494 abc ±156 | 1568 de ±77 | 2089 cde ±180 | 5992 ab ±704 | 247 e ±25 | 5930 ab ±866 | 248 e ±22 | R,L,M | *<0.0001* |
| trans-beta-Ocimene | 1039 | 40 a ±6 | 16 ab ±2 | 33 a ±0 | nd | nd | 27 ab ±7 | nd | 45 a ±23 | nd | L,M | *0.00* |
| α-Terpinene | 1056 | 408 b ±23 | 615 a ±35 | 322 bc ±0 | 74 de ±12 | 142 d ±8 | 277 c ±18 | 55 de ±1 | 392 b ±61 | nd | R,L,M | *<0.0001* |
| Geijerene | 1136 | 208 a ±128 | 305 a ±23 | 322 a ±0 | 295 a ±49 | 315 a ±126 | 444 a ±101 | 204 a ±22 | 469 a ±20 | 285 a ±177 | L,M | *0.18* |
| *Total* | | *8846 a* | *5905 b* | *6159 ab* | *2162 c* | *3037 c* | *7704 ab* | *648 c* | *7911 ab* | *637 c* | | *<0.0001* |
| ***Monoterpenoid alcohols*** | | | | | | | | | | | | |
| Linalool | 1100 | 1347 c ±70 | 1561 b ±29 | 1302 c ±0 | 945 d ±46 | 787 e ±6 | 777 e ±35 | 182 f ±9 | 1751 a ±3 | 784 e ±27 | R,L,M | *<0.0001* |
| ***Oxygenated monoterpenes*** | | | | | | | | | | | | |
| 2-methoxy-p-Cymene | 1237 | nd | nd | nd | nd | nd | nd | 323 a ±53 | nd | nd | L,M | *<0.0001* |
| Fenchone | 1084 | 45 c ±2 | nd | 101 a ±0 | 15 d ±2 | 48 c ±3 | nd | nd | 70 b ±0 | nd | R,L,M | *<0.0001* |
| Dihydrocarvone | 1194 | nd | nd | nd | nd | nd | 134 b ±34 | nd | 261 a ±11 | nd | L,M | *<0.0001* |
| p-Cuminic aldehyde | 1238 | 623 ab ±258 | 759 a ±3 | 795 a ±52 | 294 bc ±169 | nd | nd | nd | nd | nd | L,M | *<0.0001* |

## Supplementary Tables

| | | | | | | | | | | | | |
|---|---|---|---|---|---|---|---|---|---|---|---|---|
| D-Carvone | 1243 | 2632 b ±565 | 1304 bc ±6 | 2711 b ±125 | 1995 bc ±367 | 2628 b ±321 | 2412 b ±328 | nd | 2832 b ±216 | 6245 a ±297 | L,M | *0.00* |
| Geranyl butanoate | 1388 | 142 b ±0 | 149 b ±0 | 131 c ±0 | nd | nd | 51 d ±5 | nd | 216 a ±4 | nd | L,M | *<0.0001* |
| *Total* | | *3442 b* | *2213 bc* | *3738 ab* | *2304 bc* | *2676 bc* | *2596 bc* | *323 c* | *3379 b* | *6245 a* | | *0.00* |
| ***Phenylpropanoid derivatives*** | | | | | | | | | | | | |
| 1-Methoxy-4-[(E)-2-methoxyethenyl]benzene | 1493 | 922 bc ±10 | 747 c ±6 | 990 bc ±0 | 1301 a ±54 | 221 d ±58 | 892 bc ±121 | 1100 ab ±44 | 856 bc ±45 | 424 d ±107 | L,M | *<0.0001* |
| cis-Anethole | 1253 | 15824 b ±608 | 15108 b ±805 | 14806 b ±0 | 17535 b ±934 | 67647 a ±996 | 17281 b ±695 | 13798 b ±323 | 21808 b ±824 | 24163 b ±788 | L,M | *<0.0001* |
| p-Anisaldehyde | 1266 | 2747 b ±142 | 576 b ±116 | 754 b ±0 | 1875 b ±586 | 4395 b ±177 | 1066 b ±692 | 2044 b ±19 | 2870 b ±583 | 14399 a ±600 | R,L,M | *0.00* |
| trans-Anethole† (mg/L) | 1305 | 1312 ef ±27 | 1364 de ±18 | 1416 cd ±3 | 2126 a ±8 | 1977 b ±6 | 1267 f ±34 | 1075 g ±9 | 1457 c ±17 | 1259 f ±5 | R,L,M | *<0.0001* |
| Chavicol | 1346 | nd | nd | nd | nd | 46 a ±11 | nd | nd | nd | nd | R,L,M | *<0.0001* |
| 1-Methoxy-4-(1-methylpropyl)-benzene | 1361 | nd | nd | 46 ab ±0 | nd | nd | nd | nd | 55 a ±15 | 32 b ±0 | L,M | *<0.0001* |
| 3,5-Dimethyl-2-(1-phenylethyl)phenol | 1366 | 229 de ±17 | 240 de ±0 | 271 cde ±0 | 396 a ±13 | 351 ab ±23 | 299 bc ±16 | 275 cd ±10 | 216 ef ±1 | 164 f ±23 | L,M | *<0.0001* |
| 4-Methoxyphenyl-2-propanone | 1385 | 676 b ±294 | 594 b ±163 | 750 b ±0 | 661 b ±61 | 1207 b ±734 | 460 b ±15 | 601 b ±204 | 979 b ±693 | 3946 a ±967 | L,M | *0.00* |
| 2-Allyl-1,4-dimethoxybenzene | 1399 | 75 cd ±3 | 58 d ±2 | 74 cd ±0 | 158 a ±4 | 155 a ±8 | 124 ab ±28 | 101 bc ±5 | 73 cd ±3 | 75 cd ±4 | L,M | *<0.0001* |
| o-Methyleugenol | 1408 | 78 cd ±4 | 52 d ±4 | 71 cd ±0 | 294 a ±9 | 209 b ±43 | 137 bc ±6 | 73 cd ±4 | 87 cd ±9 | 100 cd ±30 | R,L,M | *<0.0001* |
| 4-Methoxyphenyl-1-propanone | 1450 | nd | nd | nd | 94 b ±0 | 88 b ±24 | nd | nd | 70 bc ±47 | 464 a ±33 | L,M | *<0.0001* |
| 2-Phenyl-2-pentene | 1517 | 60 cd ±27 | 137 b ±18 | 113 bc ±0 | 226 a ±6 | 81 bcd ±8 | nd | 100 bcd ±21 | 90 bcd ±14 | 47 de ±12 | L,M | *<0.0001* |
| Dill apiol | 1626 | 68 a ±3 | 16 de ±3 | 33 cd ±0 | nd | nd | 47 bc ±6 | 0 e ±0 | 59 ab ±13 | nd | L,M | *<0.0001* |
| 2-(1-E-propenyl)-4-methoxyphenyl 2-methylbutanoate | 1845 | 1364 b ±276 | 957 b ±63 | 1058 b ±0 | 416 c ±12 | 2 c ±3 | 1229 b ±162 | 2702 a ±170 | 1179 b ±53 | nd | L,M | *<0.0001* |
| p-Vinylanisole | 1149 | 60 a ±14 | 56 a ±3 | 60 a ±0 | 66 a ±18 | 219 a ±203 | 87 a ±24 | nd | nd | nd | L,M | *0.17* |
| Estragole† (mg/L) | 1198 | 29 bc ±3 | 26 bc ±2 | 27 bc ±3 | 56 a ±4 | 51 a ±2 | 20 cd ±1 | 16 d ±1 | 34 b ±3 | 16 d ±2 | R,L,M | *<0.0001* |
| p-Methoxycinnamaldehyde | 1566 | nd | nd | nd | nd | nd | nd | nd | 50 b ±2 | 183 a ±39 | L,M | *<0.0001* |
| *Total (mg/L)* | | *1362 ef* | *1409 de* | *1461 cd* | *2204 a* | *2103 b* | *1309 f* | *1111 g* | *1520 c* | *1320 f* | | *<0.0001* |
| ***Sesquiterpene hydrocarbons*** | | | | | | | | | | | | |
| δ-Elemene | 1339 | 152 cd ±39 | 165 bcd ±79 | 258 abc ±0 | 385 a ±23 | 203 bcd ±45 | 320 ab ±56 | 85 d ±14 | 214 bcd ±27 | 75 d ±38 | R,L,M | *0.00* |
| α-Longipinene | 1348 | 41 ab ±2 | 31 b ±11 | 35 b ±0 | 57 ab ±5 | 50 ab ±1 | 79 a ±15 | 30 b ±1 | 38 ab ±7 | 46 ab ±23 | L,M | *0.02* |
| α-Ylangene | 1370 | 35 abc ±12 | 38 abc ±14 | 44 ab ±0 | 65 a ±5 | 52 a ±0 | 67 a ±13 | 8 c ±12 | 33 abc ±7 | 16 bc ±0 | L,M | *0.00* |
| β-Elemene | 1391 | 120 bc ±15 | 124 bc ±36 | 136 bc ±0 | 288 a ±9 | 186 b ±7 | 188 b ±29 | 81 c ±17 | 140 bc ±11 | 68 c ±26 | L,M | *<0.0001* |
| β-Ylangene | 1418 | nd | 30 b ±16 | 33 b ±0 | 101 a ±2 | 26 b ±36 | 21 b ±30 | 17 b ±1 | 45 ab ±6 | 19 b ±5 | L,M | *0.01* |
| β-Gurjunene | 1427 | nd | nd | nd | 72 a ±6 | 18 b ±25 | nd | nd | nd | nd | L,M | *0.00* |
| γ-Elemen | 1437 | 135 bc ±2 | 86 c ±9 | 113 bc ±0 | 182 bc ±2 | 751 a ±106 | 240 b ±14 | 78 c ±13 | 90 c ±2 | 66 c ±16 | L,M | *<0.0001* |
| α-Himachalene | 1445 | 362 bcd ±80 | 344 bcd ±114 | 404 bc ±0 | 879 a ±48 | 534 b ±48 | 594 b ±96 | 233 cd ±52 | 377 bc ±56 | 113 d ±25 | L,M | *<0.0001* |

Supplementary Tables

| | | | | | | | | | | | | |
|---|---|---|---|---|---|---|---|---|---|---|---|---|
| cis-β-Farnesene | 1461 | 68 bcd ±7 | 72 bcd ±32 | 95 bc ±0 | 178 a ±20 | 99 abc ±22 | 113 ab ±14 | 62 bcd ±39 | 25 cd ±0 | 0 d ±0 | L,M | *0.00* |
| 8,9-dehydro-Neoisolongifolene | 1468 | 73 cd ±5 | 165 ab ±39 | 127 bc ±0 | 211 a ±4 | 71 cd ±7 | 119 bc ±6 | 112 bc ±25 | 105 bc ±5 | 37 d ±11 | L,M | *<0.0001* |
| γ-Himachalene | 1475 | 4232 bc ±717 | 3892 bcd ±862 | 4773 bc ±0 | 9830 a ±311 | 5672 bc ±717 | 6091 b ±891 | 3163 cd ±535 | 4728 bc ±670 | 1452 d ±131 | R,L,M | *<0.0001* |
| Germacrene D | 1479 | 315 bc ±46 | 378 b ±108 | 423 b ±0 | 812 a ±2 | 255 bc ±20 | 405 b ±58 | 130 c ±28 | 384 b ±66 | 124 c ±27 | L,M | *<0.0001* |
| α-Elemene | 1481 | 212 bc ±34 | 207 bc ±53 | 260 bc ±0 | 569 a ±19 | 288 b ±0 | 267 bc ±42 | 151 cd ±21 | 226 bc ±34 | 59 d ±15 | L,M | *<0.0001* |
| ar-Curcumene | 1484 | 319 c ±43 | 385 c ±111 | 510 abc ±0 | 893 ab ±100 | 539 abc ±196 | 579 abc ±135 | 281 c ±85 | 473 bc ±35 | 945 a ±174 | L,M | *0.00* |
| α-Zingiberene | 1496 | 535 bc ±176 | 537 bc ±210 | 880 b ±0 | 1452 a ±101 | 602 bc ±166 | 985 ab ±215 | 182 c ±90 | 594 bc ±77 | 121 c ±5 | R,L,M | *0.00* |
| β-Himachalene | 1497 | 251 bc ±3 | 240 bc ±58 | 206 c ±0 | 548 a ±42 | 329 b ±23 | 232 bc ±27 | 195 c ±8 | 225 bc ±20 | 85 d ±1 | L,M | *<0.0001* |
| δ-Cadinene | 1506 | 48 bcd ±9 | 49 bcd ±18 | 58 bcd ±0 | 160 a ±23 | 64 bc ±9 | 79 b ±22 | 25 cd ±6 | 48 bcd ±7 | 8 d ±3 | L,M | *<0.0001* |
| β-Bisabolene | 1509 | 261 b ±85 | 252 b ±87 | 395 ab ±0 | 794 a ±78 | 435 ab ±162 | 502 ab ±108 | 162 b ±38 | 348 b ±10 | 351 b ±219 | L,M | *0.01* |
| β-Sesquiphellandrene | 1525 | 136 b ±9 | 133 b ±32 | 211 b ±0 | 407 a ±46 | 241 b ±70 | 247 b ±59 | 134 b ±8 | 190 b ±35 | 100 b ±11 | L,M | *0.00* |
| γ-Dehydro-ar-himachalene | 1529 | nd | nd | nd | nd | 325 a ±116 | 0 b ±0 | 47 b ±14 | nd | 135 b ±13 | L,M | *0.00* |
| α-Calacorene | 1542 | nd | 31 abc ±8 | 46 abc ±0 | 79 ab ±1 | 108 a ±44 | 53 abc ±34 | 26 bc ±7 | 46 abc ±12 | 39 abc ±2 | L,M | *0.01* |
| β-Vatirenene | 1629 | 140 ab ±16 | 93 bc ±1 | 135 ab ±0 | 245 a ±8 | 145 ab ±62 | 42 bc ±22 | 215 a ±14 | 91 bc ±0 | 0 c ±0 | L,M | *0.00* |
| α-Cuprenene | 1643 | 92 ab ±6 | 49 ab ±17 | 94 ab ±0 | 125 a ±5 | 96 ab ±49 | 24 b ±16 | 107 a ±4 | 94 ab ±2 | 104 ab ±12 | L,M | *0.02* |
| *Total* | | *7528 bcd* | *7301 bcd* | *9235 bc* | *18331 a* | *11089 b* | *11340 b* | *5525 cd* | *8515 bcd* | *3966 d* | | *<0.0001* |
| ***Sesquiterpene alcohols*** | | | | | | | | | | | | |
| Cedran-8-ol | 1646 | 28 cde ±11 | 16 e ±5 | 31 bcde ±1 | 52 ab ±8 | 55 a ±3 | 25 de ±6 | 47 abc ±4 | 30 bcde ±4 | 41 abcd ±0 | L,M | *0.00* |
| cis-Farnesol | 1685 | nd | nd | nd | nd | nd | nd | 50 a ±1 | nd | nd | R,L,M | *<0.0001* |
| *Total* | | *28 de* | *16 e* | *31 cde* | *52 bc* | *55 b* | *25 de* | *97 a* | *30 cde* | *41 bcd* | | *<0.0001* |
| ***Aromadendrane sesquiterpenoids*** | | | | | | | | | | | | |
| Spathulenol | 1576 | nd | 38 ab ±5 | 34 ab ±2 | 87 ab ±0 | 44 ab ±62 | 39 ab ±14 | 109 a ±37 | nd | nd | L,M | *0.01* |
| Isospathulenol | 1594 | 33 a ±47 | 68 a ±16 | 81 a ±3 | 121 a ±51 | 100 a ±53 | 63 a ±11 | 152 a ±17 | 81 a ±16 | 108 a ±40 | L,M | *0.13* |
| *Total* | | *33 b* | *106 ab* | *115 ab* | *208 ab* | *144 ab* | *103 ab* | *261 a* | *81 ab* | *108 ab* | | *0.04* |
| ***Volatile Phenols*** | | | | | | | | | | | | |
| 2,6-Dimethyl-phenol | 933 | nd | nd | nd | nd | nd | nd | nd | 15 a ±2 | 0 b ±0 | R,L,M | *<0.0001* |
| 2-Hydroxy-4-methoxyacetophenone | 1460 | nd | nd | nd | nd | nd | nd | nd | 33 b ±7 | 723 a ±9 | L,M | *<0.0001* |
| 2-Hydroxypropyl-4-methyl-phenol | 1552 | nd | nd | nd | nd | nd | nd | nd | 31 b ±25 | 318 a ±28 | L,M | *<0.0001* |
| *Total* | | | | | | | | | *79 b* | *1041 a* | | *<0.0001* |
| ***Higher Alcohols*** | | | | | | | | | | | | |
| 3-Methyl-1-butanol+2-Methyl-1-butanol | 751 | 546 bc ±9 | 670 bc ±45 | 475 c ±0 | 1392 a ±54 | 1320 a ±121 | 781 bc ±114 | 597 bc ±58 | 485 c ±84 | 1043 ab ±315 | R,L,M | *0.00* |
| ***Aldehydes*** | | | | | | | | | | | | |

Supplementary Tables

| | | | | | | | | | | | | |
|---|---|---|---|---|---|---|---|---|---|---|---|---|
| 2-Diethoxymethyl-3-methyl-butyraldehyde | 957 | nd | nd | nd | nd | nd | nd | nd | 30 a ±8 | nd | L,M | *<0.0001* |
| Nonanal | 1106 | nd | 53 ab ±2 | 66 ab ±3 | 59 ab ±11 | 63 ab ±2 | nd | 35 b ±1 | 106 ab ±35 | 243 a ±150 | L,M | *0.03* |
| Decanal | 1175 | nd | 71 a ±2 | nd | nd | nd | nd | nd | nd | nd | L,M | *<0.0001* |
| *Total* | | | *123 ab* | *66 ab* | *59 ab* | *63 ab* | | *35 b* | *137 ab* | *243 a* | | *0.02* |
| ***Alkenes*** | | | | | | | | | | | | |
| 2,3-dimethyl-2-Butene | 717 | 148 a ±33 | 143 ab ±25 | 142 ab ±8 | 92 abc ±9 | 59 c ±5 | 85 bc ±3 | 104 abc ±1 | 71 c ±6 | 102 abc ±13 | L,M | *0.00* |
| ***Furans*** | | | | | | | | | | | | |
| 2-pentyl-Furan | 991 | 147 a ±5 | 127 a ±15 | 88 abc ±6 | nd | 34 bc ±48 | 93 ab ±10 | 28 bc ±2 | 137 a ±47 | nd | L,M | *0.00* |
| **General Total (mg/L)** | | **1387 ef** | **1429 e** | **1487 d** | **2235 a** | **2125 b** | **1337 f** | **1121 g** | **1549 c** | **1340 f** | | ***<0.0001*** |

Values for concentration are the mean ± standard deviation (n = 3); †results are from direct injection to GC-FID. LRI, Kovats index value determined in HP-5MS capillary column (30 m x 0.25 mm x 0.25 µm); nd, not detected; ID; Identification. M: identification by comparison with the mass spectrum from NIST library. L: identification by comparison with data from previous literature. R: identification with the injection of reference compounds. *p*: Significance at which means differ as shown as analysis of variance, a–g Different letters within the same groups are significantly different by Tukey's multiple range test ($p < 0.05$).

# Supplementary Tables

Table S3. Attributes' mean scores for each rakı sample given by trained assessors in the descriptive analysis

| **Attributes** | **1R** | | **2R** | | **3R** | | **4R** | | **5R** | | **6R** | | **7R** | | **8R** | | **9R** | | *P* |
|---|---|---|---|---|---|---|---|---|---|---|---|---|---|---|---|---|---|---|---|
| Color | 5.1 | bc | 7.2 | abc | 7.7 | abc | 11.6 | a | 8.9 | ab | 7.3 | abc | 7.0 | abc | 3.6 | c | 6.5 | bc | **0.001** |
| Pale Yellow | 0.1 | c | 0.1 | c | 0.1 | c | 0.2 | c | 0.2 | c | 0.2 | c | 0.2 | c | 6.0 | a | 2.1 | b | **<.0001** |
| Visual Coating | 4.6 | b | 5.2 | ab | 4.8 | b | 10.8 | a | 8.9 | ab | 5.5 | ab | 5.5 | ab | 8.2 | ab | 6.8 | ab | **0.011** |
| Pungent | 6.0 | a | 7.2 | a | 7.4 | a | 6.4 | a | 5.1 | a | 6.0 | a | 7.1 | a | 7.8 | a | 6.6 | a | 0.637 |
| Fresh Aniseed | 9.0 | a | 8.9 | a | 10.4 | a | 8.3 | a | 8.6 | a | 9.2 | a | 8.5 | a | 7.7 | a | 6.6 | a | 0.584 |
| Boiled Aniseed | 3.4 | a | 4.2 | a | 3.4 | a | 7.1 | a | 5.6 | a | 3.9 | a | 4.1 | a | 6.6 | a | 5.4 | a | 0.136 |
| Suma Odor | 8.3 | a | 7.8 | a | 7.2 | a | 9.0 | a | 8.1 | a | 9.9 | a | 8.4 | a | 8.3 | a | 7.4 | a | 0.383 |
| Grape | 3.6 | a | 4.4 | a | 5.5 | a | 4.7 | a | 4.3 | a | 5.1 | a | 4.8 | a | 5.7 | a | 5.4 | a | 0.962 |
| Raisin | 5.7 | a | 6.0 | a | 2.6 | a | 5.5 | a | 6.6 | a | 5.7 | a | 6.1 | a | 4.1 | a | 5.2 | a | 0.504 |
| Mastic | 3.7 | a | 5.1 | a | 4.6 | a | 6.0 | a | 5.9 | a | 4.4 | a | 2.6 | a | 3.8 | a | 4.6 | a | 0.117 |
| Menthol | 6.0 | a | 7.9 | a | 7.0 | a | 6.0 | a | 7.1 | a | 6.1 | a | 6.6 | a | 6.3 | a | 6.5 | a | 0.934 |
| Resin/Fresh | 4.5 | a | 7.3 | a | 6.3 | a | 4.7 | a | 4.6 | a | 4.8 | a | 5.9 | a | 5.5 | a | 4.9 | a | 0.260 |
| Spicy | 7.2 | a | 8.7 | a | 7.6 | a | 8.6 | a | 7.1 | a | 7.0 | a | 7.2 | a | 8.9 | a | 8.3 | a | 0.836 |
| Black Pepper | 4.4 | a | 3.1 | a | 2.1 | a | 3.7 | a | 2.9 | a | 3.4 | a | 3.1 | a | 4.2 | a | 3.2 | a | 0.740 |
| Coriander | 6.2 | abc | 5.7 | abc | 6.6 | ab | 5.6 | abc | 5.6 | abc | 7.0 | a | 4.0 | c | 6.3 | abc | 4.3 | bc | **0.007** |
| Clove | 1.1 | a | 3.3 | a | 2.6 | a | 1.8 | a | 3.1 | a | 2.2 | a | 2.0 | a | 2.2 | a | 1.9 | a | 0.336 |
| Bitter Almond | 1.1 | a | 0.9 | a | 2.4 | a | 1.1 | a | 1.9 | a | 1.7 | a | 1.8 | a | 2.6 | a | 2.1 | a | 0.593 |
| Dried Flower | 4.9 | a | 1.5 | a | 2.7 | a | 4.0 | a | 2.9 | a | 3.6 | a | 3.4 | a | 4.8 | a | 4.6 | a | 0.148 |
| Wood/Oak | 0.8 | b | 0.5 | b | 0.7 | b | 0.2 | b | 0.5 | b | 0.7 | b | 0.6 | b | 3.5 | a | 2.2 | ab | **0.001** |
| Sweetness | 7.6 | a | 7.6 | a | 6.6 | a | 8.8 | a | 8.4 | a | 8.0 | a | 7.1 | a | 8.1 | a | 6.9 | a | 0.714 |
| Bitterness | 3.2 | a | 5.1 | a | 5.2 | a | 5.2 | a | 2.4 | a | 3.2 | a | 2.7 | a | 2.9 | a | 4.4 | a | 0.072 |
| Astringency | 1.8 | b | 3.4 | ab | 3.2 | ab | 3.4 | ab | 2.9 | ab | 2.1 | b | 1.6 | b | 4.6 | ab | 5.8 | a | **0.003** |
| Body | 5.2 | b | 6.0 | b | 6.5 | b | 11.0 | a | 7.5 | ab | 6.3 | b | 4.1 | b | 6.6 | b | 6.7 | b | **0.0001** |
| Alcohol Burning | 5.6 | a | 5.4 | a | 6.4 | a | 6.6 | a | 5.2 | a | 5.2 | a | 6.6 | a | 5.7 | a | 4.9 | a | 0.944 |
| Creamy | 4.6 | abc | 4.2 | bc | 2.7 | c | 8.6 | a | 7.1 | ab | 5.4 | abc | 3.6 | bc | 6.7 | abc | 5.5 | abc | **0.001** |
| Throat Harshness | 4.9 | a | 4.4 | a | 7.3 | a | 6.9 | a | 5.7 | a | 6.1 | a | 4.0 | a | 4.2 | a | 5.8 | a | 0.190 |
| Complexity | 4.6 | a | 6.9 | a | 5.4 | a | 8.4 | a | 7.3 | a | 5.9 | a | 5.2 | a | 6.5 | a | 6.5 | a | 0.235 |
| Persistency | 6.7 | a | 6.5 | a | 8.9 | a | 10.5 | a | 8.5 | a | 8.2 | a | 6.3 | a | 7.1 | a | 8.7 | a | **0.045** |
| p-Suma | 7.8 | a | 7.6 | a | 7.2 | a | 8.3 | a | 8.1 | a | 8.3 | a | 7.9 | a | 8.3 | a | 8.3 | a | 0.941 |
| p-Grape | 5.5 | a | 4.0 | a | 5.7 | a | 6.3 | a | 3.0 | a | 3.6 | a | 4.8 | a | 4.2 | a | 5.2 | a | 0.435 |
| p-Raisin | 4.0 | a | 4.2 | a | 2.8 | a | 6.3 | a | 6.1 | a | 4.2 | a | 5.0 | a | 3.0 | a | 4.8 | a | 0.482 |
| p-Fresh Aniseed | 8.9 | a | 8.5 | a | 8.6 | a | 7.9 | a | 7.2 | a | 8.9 | a | 7.7 | a | 6.2 | a | 7.0 | a | 0.621 |
| p-Boiled Aniseed | 3.0 | a | 4.4 | a | 4.7 | a | 5.9 | a | 6.1 | a | 3.2 | a | 3.9 | a | 5.3 | a | 6.1 | a | 0.371 |
| p-Mastic | 3.8 | a | 4.5 | a | 4.6 | a | 5.5 | a | 4.1 | a | 4.1 | a | 3.5 | a | 3.0 | a | 2.9 | a | 0.540 |
| p-Menthol | 5.0 | a | 8.3 | a | 6.9 | a | 5.8 | a | 7.6 | a | 4.8 | a | 4.7 | a | 5.8 | a | 4.8 | a | 0.288 |
| p-Spicy | 5.8 | a | 5.8 | a | 5.0 | a | 8.0 | a | 7.9 | a | 6.1 | a | 5.7 | a | 7.0 | a | 7.0 | a | 0.591 |
| p-Dried Flower | 3.3 | a | 0.9 | a | 1.4 | a | 4.2 | a | 2.4 | a | 2.8 | a | 3.1 | a | 3.3 | a | 3.4 | a | 0.185 |

*p*: statistical differences were tested by two-way ANOVA; p-values in bold indicated statistically significant. abc: values in the same row with different letters differ significantly (Tukey test. $p<0.05$). P, on palate retronasal aroma.

# Supplementary Tables

Table S4. Demographic profiles of participants

| | Categories | Participants Frequencies (%) |
|---|---|---|
| Age | 18-29 | 38 |
| | 30-45 | 47 |
| | 46-65 | 15 |
| Cender | Female | 46 |
| | Male | 54 |
| Consumer frequency | rarely | 10 |
| | once a week | 10 |
| | several times a month | 42 |
| | several times a year | 38 |

Table S5. Distribution of rakı consumption frequency by age groups

| Consumer frequency \ Age | Age-18-29 | Age-30-45 | Age-46-65 | Total |
|---|---|---|---|---|
| Consumer frequency-rarely | 8 | 2 | 0 | 10 |
| Consumer frequency-once a week | 2 | 5 | 3 | 10 |
| Consumer frequency-several times a month | 12 | 21 | 9 | 42 |
| Consumer frequency-several times a year | 16 | 19 | 3 | 38 |
| Total (*N*) | 38 | 47 | 15 | 100 |

*N*, the number of consumers

# Supplementary Tables

Table S6. Counts of checked attributes by 100 panelists for CATA

| Attributes | *Counts* | *%* |
|---|---|---|
| Greyish White | 263 | 29% |
| White | 241 | 27% |
| Intense Bright White | 109 | 12% |
| Slightly Yellow | 130 | 14% |
| Low Coating | 271 | 30% |
| Medium Coating | 240 | 27% |
| High Coating | 106 | 12% |
| Pungent | 287 | 32% |
| Fresh Aniseed | 598 | 66% |
| Cooked/Boiled Aniseed | 199 | 22% |
| Fresh Grape | 178 | 20% |
| Raisin | 199 | 22% |
| Spicy | 178 | 20% |
| Mastic | 114 | 13% |
| Menthol | 150 | 17% |
| Fresh/Resinous | 89 | 10% |
| Black Pepper | 187 | 21% |
| Coriander | 224 | 25% |
| Dried Flower/Herb | 83 | 9% |
| Sweet Odor | 74 | 8% |
| Woody | 70 | 8% |
| Bitter Almond | 178 | 20% |
| Aromatic | 93 | 10% |
| Burning | 78 | 9% |
| Sweetness | 174 | 19% |
| Bitterness | 287 | 32% |
| Astringency | 260 | 29% |
| Full-body | 224 | 25% |
| Low-body | 201 | 22% |
| Harsh | 220 | 24% |
| Smooth | 119 | 13% |
| Creamy | 201 | 22% |
| Persistent | 555 | 62% |
| Throat Burning | 241 | 27% |
| P-Spicy | 187 | 21% |
| P-Black Pepper | 254 | 28% |
| P-Dried Flower /Herb | 123 | 14% |
| P-Fresh Anise | 67 | 7% |
| P-Cooked/Boiled Anise | 59 | 7% |
| P-Mastic | 217 | 24% |
| P-Coriander/Cumin | 118 | 13% |
| P-Fresh Grape | 75 | 8% |
| P-Raisin | 53 | 6% |

P, on palate retronasal aroma. %, represent the frequency of selection.
